# Active synthesis of type I collagen homotrimer in Dupuytren’s fibrosis is unaffected by anti–TNF-**α** treatment

**DOI:** 10.1172/jci.insight.175188

**Published:** 2025-05-08

**Authors:** Kate Williamson, Katie J. Lee, Emma L. Beamish, Alan Carter, Jade A. Gumbs, Gabriella Cooper, Niamh S. O’Heneghan-Yates, Lisa A. Menezes, Graham Cheung, Daniel Brown, Rob Pettitt, Brendan Geraghty, Lucy A. Bosworth, Eithne J. Comerford, Peter D. Clegg, Elizabeth G. Canty-Laird

**Affiliations:** 1 Department of Musculoskeletal and Ageing Science, Institute of Life Course and Medical Sciences, University of Liverpool, William Henry Duncan Building, Liverpool, United Kingdom.; 2 The Medical Research Council Versus Arthritis Centre for Integrated Research into Musculoskeletal Ageing (CIMA), William Henry Duncan Building, Liverpool, United Kingdom.; 3 Department of Trauma and Orthopaedics, Liverpool University Hospitals NHS Foundation Trust, Liverpool, United Kingdom.; 4 Institute of Infection, Veterinary and Ecological Sciences, Leahurst Campus, University of Liverpool, Neston, United Kingdom.; 5 Department of Eye and Vision Sciences, Institute of Life Course and Medical Sciences, University of Liverpool, William Henry Duncan Building, Liverpool, United Kingdom.

**Keywords:** Cell biology, Therapeutics, Collagens, Extracellular matrix, Fibrosis

## Abstract

Dupuytren’s disease is a common fibroproliferative disease of the palmar fascia of the hand, with advanced cases treated surgically. Anti-TNF injection has undergone phase 2 trials and may be effective in slowing early-stage disease progression. Here we sought to determine how new synthesis of type I collagen in Dupuytren’s differs from normal palmar fascia samples and to analyze the role of TNF in aberrant collagen synthesis. Model nonfibrotic but fibrous connective tissues were used to analyze active type I collagen protein synthesis in development, aging, and degenerative disease, where it was restricted to early development and ruptured tissue. Dupuytren’s tissue was shown to actively synthesize type I collagen, including abnormal type I collagen homotrimer. TNF-α reduced *COL1A2* gene expression only in the presence of serum in 2D cell culture and had opposing effects on collagen protein production in the presence or absence of serum. TNF-α had only limited effects in 3D tendon–like constructs. Anti-TNF did not reduce type I collagen synthesis in 3D tendon–like constructs or prevent type I collagen homotrimer synthesis in Dupuytren’s tissue. Hence, modulation of the TNF-α pathway in Dupuytren’s disease is unlikely to prevent the pathological collagen accumulation that is characteristic of fibrosis.

## Abstract 2

Over-production of an abnormal form of the most abundant collagen, in the hand disease Dupuytren’s fibrosis, was unaffected by treatment with the biologic anti-inflammatory anti-TNF.

## Graphical abstract

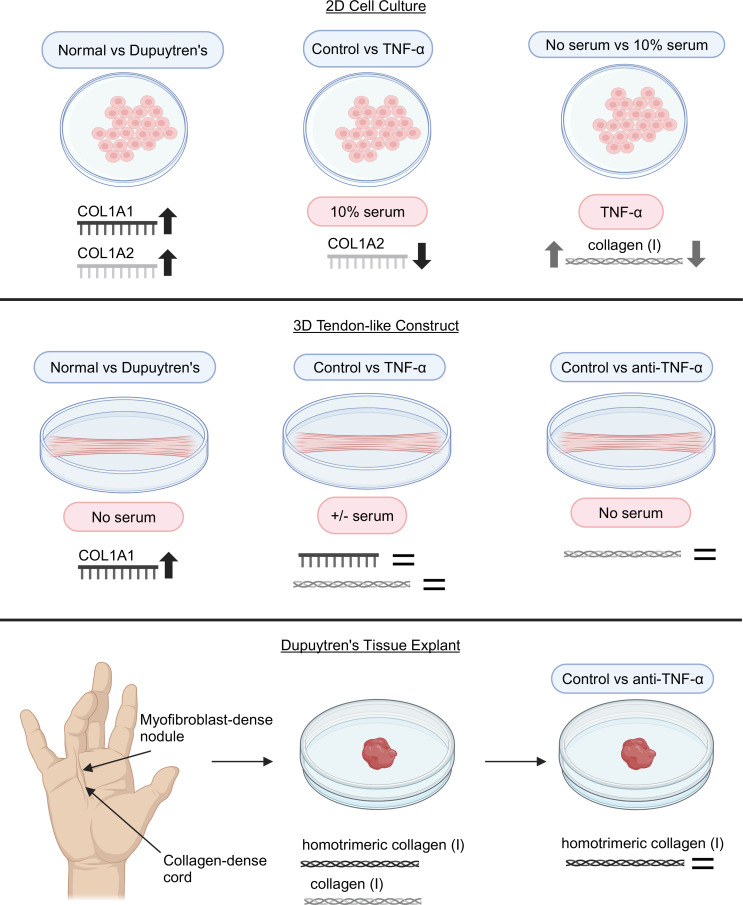

## Introduction

Fibrillar collagens, particularly type I, are the major structural component of fibrous connective tissue including palmar fascia (aponeurosis), tendon, ligament, and fibrotic tissue. In fibrous tissue string-like fibrils comprise arrays of collagen molecules. Excessive accumulation of fibrillar collagens impedes normal tissue function resulting in particularly poor outcomes in cardiac, pulmonary, kidney, and liver fibrosis (1, 2). Dupuytren’s disease is a common fibroproliferative disorder of the palmar fascia of the hand, which has sex, age, geographic, and racial differences; it is most prevalent in, but not restricted to, older men of Northern European descent (3). The etiology of Dupuytren’s is complex involving genetics; an autosomal dominance pattern with varying penetrance, links to other diseases such as diabetes, epilepsy, and liver disease as well as environmental factors including alcohol intake and smoking (4).

The formation of fibrous tissue under the skin can cause patients with Dupuytren’s localized pain and discomfort whilst disease progression may prevent the digits from straightening, producing fixed flexion contractures. The disease severity is commonly measured by the degree of joint contraction (which can be monitored by Tubiana staging), the number of digits involved, and the presence of disease outside the hand (i.e., Ledderhose disease in the foot or Peyronie’s disease in the penis) (5, 6). Treatment options include surgery, fasciectomy (excising the diseased tissue) and dermofasciectomy (excising the disease tissue and overlying skin), percutaneous needle aponeurotomy (dividing the diseased tissue), and collagenase treatment (prior to withdrawal from several jurisdictions) (7). Each treatment results in varying success, complications, and recurrence rates (8). In patients with advanced disease and debilitating contractures, the gold standard treatment is open limited fasciectomy followed by physiotherapy to encourage improved range of movement (9). Surgical complication rates increase with disease severity and range between 3% and 50% (10), while recurrence in the same finger/thumb or disease progression in other digits is common (8%–54%) (9).

The fibrotic tissue in Dupuytren’s can comprise a highly cellular “nodule” region, which is thought to represent an active stage in the tissue pathogenesis and a “cord” region, consisting of mature fibrillar collagen (11). Disease pathogenesis has been divided into proliferative, involutional (contracting), and residual stages, with progressively decreasing cellularity and increasing alignment along directional lines of tension (12). Type III collagen is present at very low levels in normal palmar fascia but is abundant in Dupuytren’s tissue (13). However, the proportion of type III collagen relative to total collagen decreases through the stages of disease progression from > 35% to < 20% in the residual stage (14). Myofibroblasts are abundant in nodules, display persistent α-smooth muscle actin (αSMA) expression, and are responsible for both matrix deposition and contraction. Bidirectional actin-fibronectin interactions (15) result in the progressive tensioning of collagen fibers and a concurrent increase in total flexion deformity (16). Other cell types implicated in the initiation or progression of Dupuytren’s fibrosis include embryonic stem cells, mesenchymal stem/stromal cells (MSCs), fibrocytes, and immune cell populations (11, 17).

Analysis of mRNA expression profiles in isolated Dupuytren’s fibroblasts found increased collagen and extracellular matrix (ECM) mRNAs (18), while a loss of collagen-regulating microRNAs (miRs) was identified in Dupuytren’s tissue (19). A weighted gene coexpression network analysis and functional enrichment analysis of Dupuytren’s transcriptomic datasets found gene ontology terms for ECM and collagen in ECM organization, ECM-receptor interaction and collagen catabolic process (20). Type I collagen is the major ECM component of fibrotic Dupuytren’s tissue, and type I collagen molecules are predominantly (α1)_2_(α2)_1_ heterotrimers derived from the polypeptide gene products of the *COL1A1* and *COL1A2* genes. However, abnormal collagen (α1)_3_ homotrimer has been identified in Dupuytren’s tissue (21). Type I collagen homotrimers are resistant to proteolytic degradation by matrix metalloproteinases (MMPs) (22) and may therefore skew the balance between type I collagen synthesis and degradation, impeding the resolution of fibrosis.

A systematic review of Dupuytren’s disease ‘omics’ studies highlighted alterations in collagen and ECM gene expression as well as genomic and transcriptomic studies implicating the TGF-β and Wnt signaling pathways in disease pathogenesis (23). TNF at 0.1 ng/mL was found to induce cell contractility, to increase αSMA mRNA and protein, and to increase *COL1A1* mRNA in palmar fibroblasts, from patients with Dupuytren’s: effects that could be reversed with the Wnt pathway inhibitor lithium chloride at 10 mM (24). Conversely, anti-TNF reduced contractility, led to disassembly of the actin cytoskeleton, reduced αSMA mRNA and protein, and decreased *COL1A1* mRNA in Dupuytren’s myofibroblasts derived from nodular tissue. Subsequently anti-TNF therapy (adalimumab) in patients with Dupuytren’s delivered by intranodular injection of a 40 mg dose was found to reduce αSMA and type I procollagen protein production (as an N-propeptide assay), though not *COL1A1* gene expression, or total collagen, in nodules obtained during surgery 2 weeks after injection (25). A longer-term study comprising 4 injections every 3 months showed a reduction in nodule size and nodule softening (26) in patients with early-stage Dupuytren’s disease. Prior studies have shown that TNF-α decreases rather than increases *COL1A1* gene expression and collagen protein synthesis in human dermal fibroblasts at 0.1 ng/mL and higher concentrations (27, 28). *COL1A2* expression and type I collagen protein is similarly decreased with 10 ng/mL TNF-α in immortalized mouse fibroblasts, *COL1A2* expression decreased in human dermal fibroblasts (29) and Col1a1 expression decreased in rat hepatic stellate cells (30).

We hypothesized that there may be a continual production of abnormal homotrimeric type I collagen that could contribute to the recurrence of Dupuytren’s contracture following medical treatment. While TNF has been identified as a potential therapeutic target in Dupuytren’s disease (24), information on the response of Dupuytren’s cells and tissues to TNF-α in terms of collagen protein synthesis and production of normal versus abnormal type I collagen is currently lacking. This information is critically important given that type I collagen protein is, by definition, the major constituent of fibrotic tissue.

In this study, we utilized metabolic labeling approaches in combination with 1D gel electrophoresis to analyze type I collagen synthesis in fibrotic Dupuytren’s tissue and in nonfibrotic, fibrous connective tissue. The study aimed to determine how fibrotic collagen differs from that produced in normal fibrous tissue and how type I collagen synthesis differs from that seen in development or in injured tissue. We evaluated the collagen biosynthetic response to TNF-α of normal palmar fascia fibroblasts, and those from Dupuytren’s nodule and cord, in both 2D cell culture and in cell-assembled collagenous 3D cultures. The effect of anti-TNF treatment on type I collagen biosynthesis was also evaluated in tissue explants and 3D cultures.

## Results

### Type I collagen homotrimer is actively produced by Dupuytren’s tissue.

**Figure 1:**
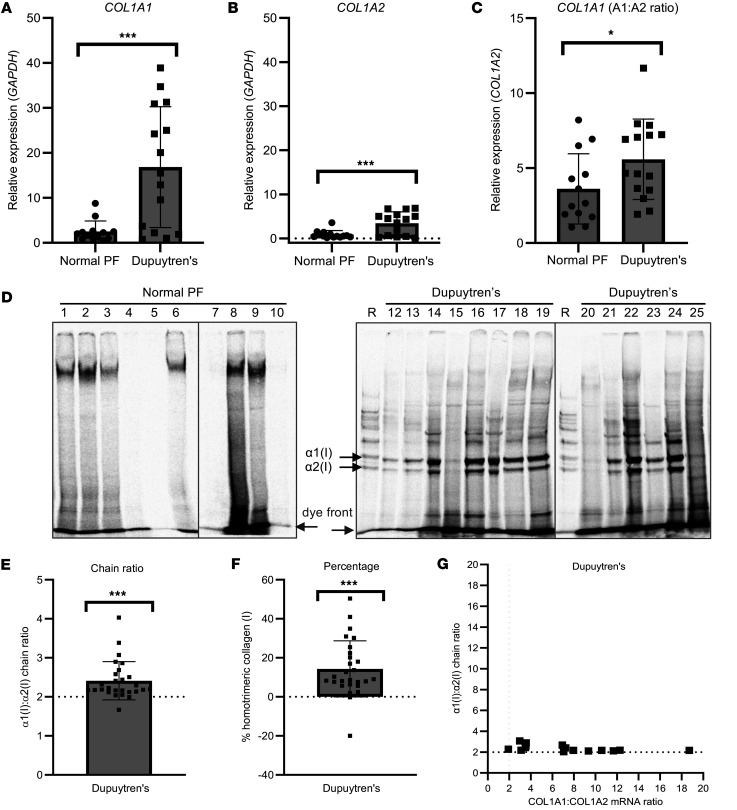
Synthesis of type I collagen homotrimer by Dupuytren’s tissue. (**A**–**C**) Analysis of *COL1A1* (**A**), *COL1A2* (**B**) and *COL1A1/COL1A2* (**C**) gene expression by qPCR in normal PF (*n* = 13) and Dupuytren’s tissue samples (*n* = 15). (**D**) Representative delayed reduction 6% SDS-PAGE gels of normal PF and Dupuytren’s tissue extracts after pulse-chase labeling with [^14^C]proline. R is a labeled type I collagen reference standard. Vertical lines delineate different gels. (**E** and **F**) The relative amounts of the labeled α1(I) and α2(I) chains in Dupuytren’s (*n* = 27) samples were quantified by densitometry and expressed as an α1(I)/α2(I) chain ratio (**E**) or converted to a percentage of homotrimeric type I collagen (**F**). (**G**) The α1(I)/α2(I) chain ratio was plotted against the *COL1A1/COL1A2* mRNA ratio for Dupuytren’s samples (*n* = 12) for which both data types were available. *P < 0.05 and ***P < 0.001 by 2-tailed Welch’s *t* test for **A**–**C** and a 1-sample *t* test for **E** and **F**.

To determine if type I collagen homotrimer is continuously synthesized in Dupuytren’s tissue, *COL1A1* and *COL1A2* gene expression was measured by quantitative PCR (qPCR), and synthesis of α-chains by radiolabeling, in Dupuytren’s samples (Supplemental Table 1; supplemental material available online with this article; https://doi.org/10.1172/jci.insight.175188DS1) and in normal palmar fascia (PF) controls (Supplemental Table 2). Expression of *COL1A1* (Figure 1A) and *COL1A2* (Figure 1B) were significantly higher in Dupuytren’s tissue (*P* = 0.001 and 0.002, respectively), indicative of new type I collagen synthesis. The relative expression of *COL1A1* as compared with *COL1A2* was also higher in Dupuytren’s tissue (*P* = 0.048) (Figure 1C), indicating increased gene transcription and/or mRNA stability of *COL1A1* as compared with *COL1A2*. There was no significant difference in the age of the Dupuytren’s and normal PF samples that were analyzed by qPCR (*P* = 0.11) (Supplemental Figure 1)

Metabolic labeling with [^14^C]proline indicated Dupuytren’s tissue explants but not normal PF controls (*n* = 22) produced newly synthesized type I collagen protein (Figure 1D). Samples derived from 27 of 31 patients with Dupuytren’s were sufficiently labeled for densitometric quantification (Supplemental Table 1). The α1(I)/α2(I) chain ratio was significantly greater than 2 (*P* < 0.001) (Figure 1E) indicative of new type I collagen homotrimer synthesis. Using the ratios for the nodule only in the analyses (discounting 3 other cord samples) produced similar results (*P* < 0.001, data not shown). The α1(I)/α2(I) ratio was converted to a percentage of homotrimeric type I collagen, indicating a mean value of 14.3% (SD ± 14.4%), with 1 sample reading 50.4% (Figure 1F). Plotting the α1(I)/α2(I) ratio against the *COL1A1/COL1A2* mRNA ratio indicated no positive linear relationship between relative mRNA and polypeptide chain ratios (Figure 1G), and the Pearson correlation coefficient was negative (*r* = –0.522, *P* = 0.046).

### Demographic factors are not associated with greater type I collagen homotrimer synthesis.

**Figure 2:**
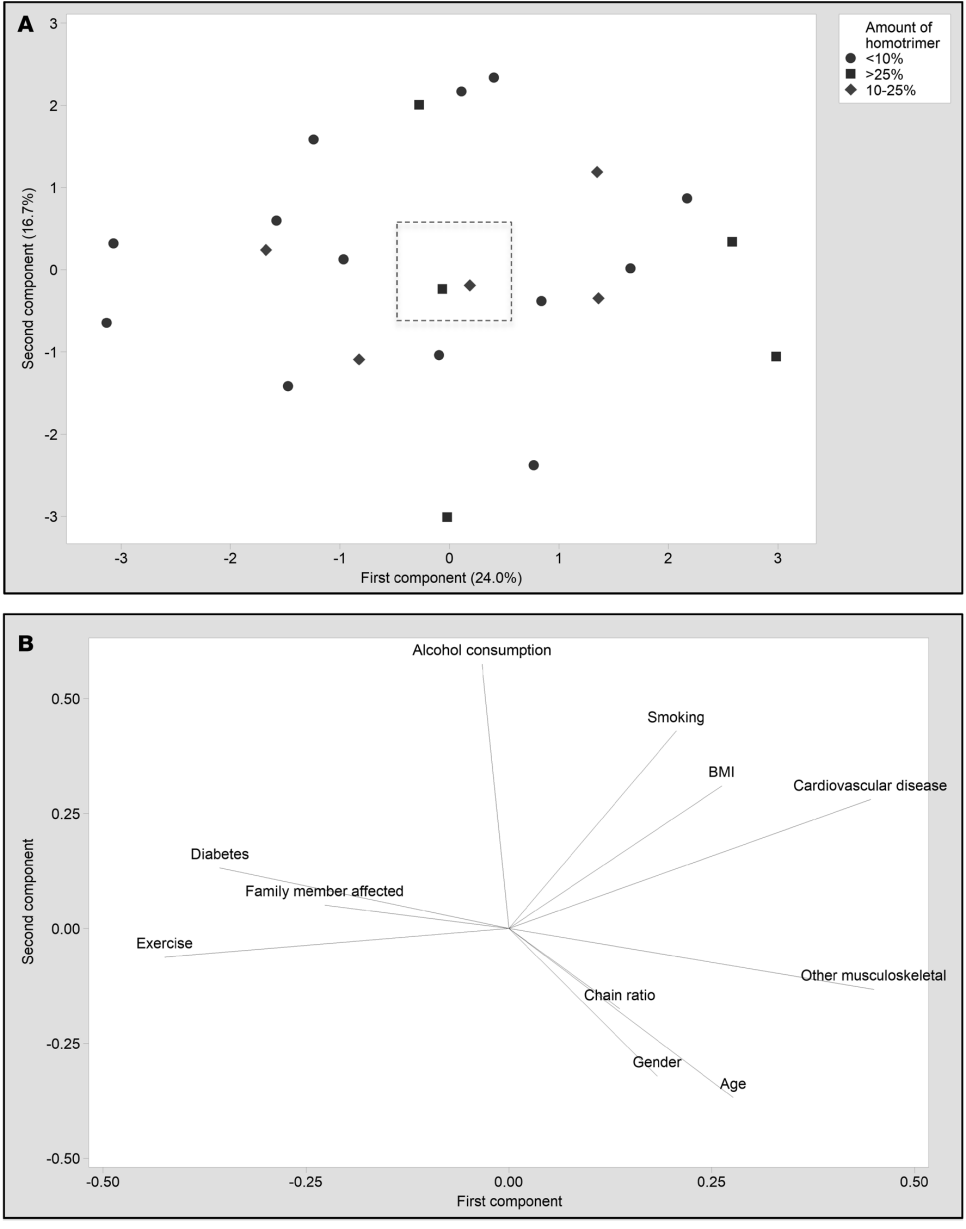
Principal component analysis of the relationship between demographic factors and the proportion of homotrimeric collagen synthesized by human Dupuytren’s surgical samples. (**A**) Score plot grouped by lower (<10%), medium (10%–25%), and higher (>25%) percentages of type I collagen homotrimer (*n* = 23). (**B**) The box indicates the relative scale on the loading plot.

To determine if particular demographic factors were associated with an increased proportion of homotrimeric type I collagen, a principal component analysis (PCA) was carried out (Figure 2). Samples producing lower (<10%), medium (10%–25%), or higher (>25%) percentages of homotrimeric type I collagen did not group together based on demographics factors (Figure 2A). Demographic factors (Figure 2B) had a minimal effect on principal component scores. To determine whether disease stage influences type I collagen homotrimer synthesis, the PCA was repeated on samples with paired disease stage, which also showed only a weak effect (Supplemental Figure 2).

### There is active type I collagen synthesis in diseased, ruptured, or fetal tissue but not in healthy adult fibrous connective tissue.

**Figure 3:**
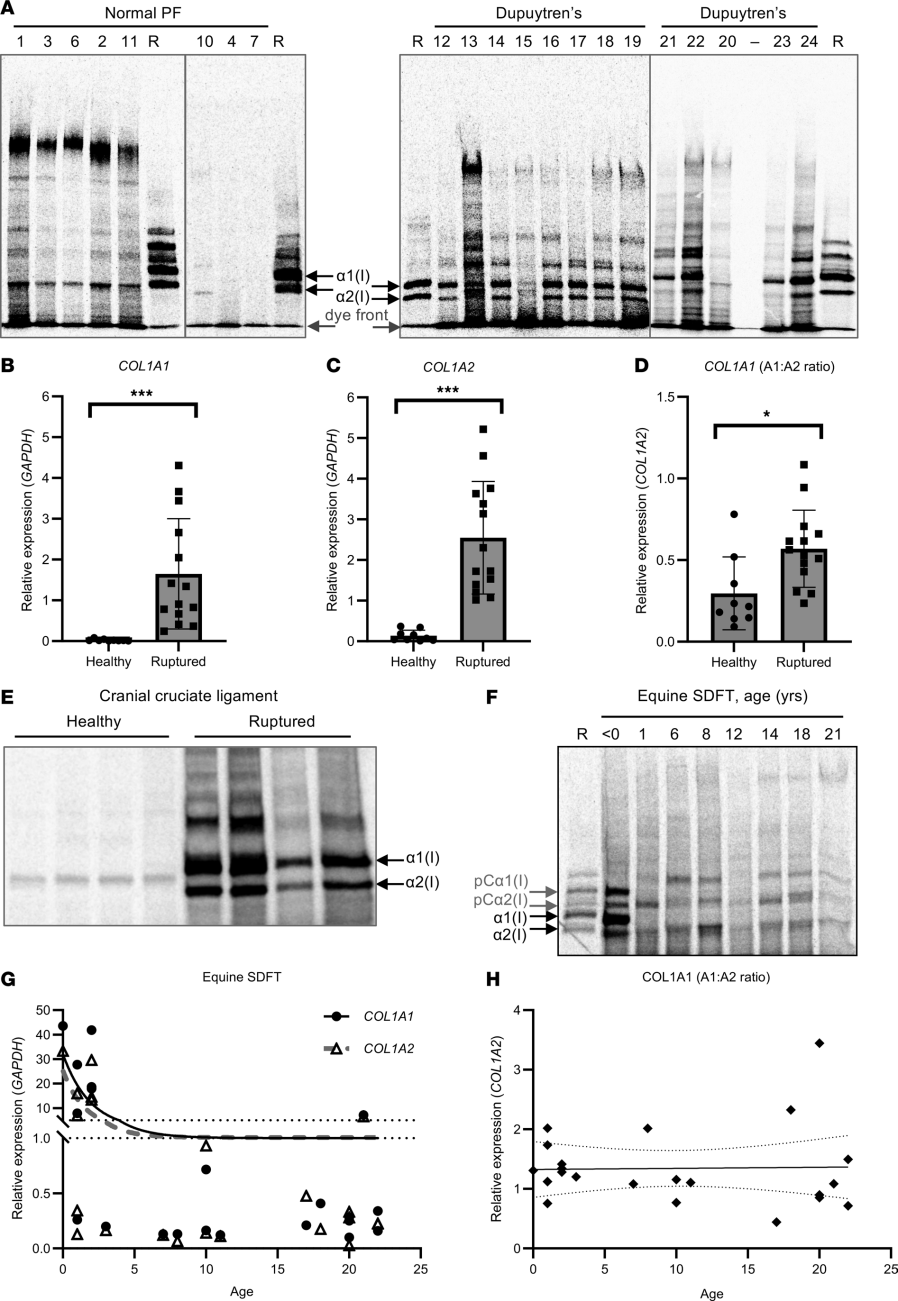
Type I collagen and proline-rich protein synthesis in tendon and ligament explants. (**A**) Representative 4% SDS-PAGE gels of reduced normal PF and Dupuytren’s tissue extracts after pulse-chase labeling with [^14^C]proline. Sample numbers relate to those shown in Figure 1D. R is a labeled type I collagen reference standard. Vertical lines delineate different gels. (**B**–**D**) Analysis of *COL1A1* (**B**), *COL1A2* (**C**) and *COL1A1/COL1A2* (**D**) gene expression by qPCR in healthy (*n* = 9) and ruptured (*n* = 14) canine cranial cruciate ligament samples. *P < 0.05 and ***P < 0.001 by Welch’s *t* test. (**E**) Representative 4% SDS-PAGE gel of reduced healthy and ruptured canine CCL extracts after pulse-chase labeling with [^14^C]proline. (**F**) Representative 4% SDS-PAGE gel of equine SDFT extracts at different ages after pulse-chase labeling with [^14^C]proline (healthy showing 4 of 15 samples [2 unlabeled], ruptured showing 4 of 24 samples). R is the collagen reference standard. (**G** and **H**) Analysis of *COL1A1* and *COL1A2* (**G**) and *COL1A1/COL1A2* (**H**) gene expression by qPCR in equine SDFT (*n* = 22) at various ages. Trend lines and 95% CI (**H**) are shown. Note: horses have an average 25- to 30-year lifespan, with 20 years being equivalent to approximately 60 human years.

No radiolabeled normal PF samples (Supplemental Table 2) were found to synthesize detectable amounts of type I collagen in explant culture, but a high molecular weight band was noted in 10 of the 22 labeled normal PF samples (Figure 1D). Under reducing conditions, a single band migrating similarly to the α2(I) chain was noted in normal PF samples and one between the α1(I) and α2(I) chain in some Dupuytren’s samples (nos. 12, 14, and 19) (Figure 3A). The identity of the labeled proteins in these bands are unknown but are expected to be collagenous, given the incorporation of labeled proline.

We considered that the age and normal status of the normal PF samples may preclude detectable levels of new type I collagen synthesis. Model nonfibrotic fibrous connective tissues were therefore studied. Analysis of canine cranial cruciate ligament (CCL) allowed a comparison between healthy fibrous tissue and those that were ruptured due to degenerative disease, while analysis of equine superficial digital flexor tendon (SDFT) facilitated a comparison across ages: fetal, young (yearling), adult (6–14 years), and old (>15 years). The relative expression of both *COL1A1* (Figure 3B) and *COL1A2* (Figure 3C) was significantly increased (both *P* < 0.001) in ruptured canine CCL as compared with healthy samples. The expression of *COL1A1* relative to *COL1A2* was higher in ruptured ligament (*P* = 0.012) (Figure 3D), although the ratio did not exceed 1.5. Ruptured ligament produced newly synthesized type I collagen while, under reducing conditions, healthy ligament produced a single band migrating between the α1(I) and the α2(I) chain (Figure 3E). Fetal SDFT produced nascent type I collagen as expected, while no postnatal SDFT samples produced nascent type I collagen, instead producing a band comigrating with α2(I) (similar to human normal PF), a higher–molecular weight band comigrating with pCα2(I) and often a band migrating between proα1(I) and pCα1(I) (Figure 3F). Gene expression of *COL1A1* and *COL1A2* in postnatal samples was highest before 2 years of age (Figure 3G), but there was no significant correlation between the *COL1A1/COL1A2* gene expression ratio and age (Figure 3H).

### TNF-α reduces COL1A2 gene expression in the presence of serum in cell culture and has opposing effects on collagen protein production in the presence or absence of serum.

**Figure 4:**
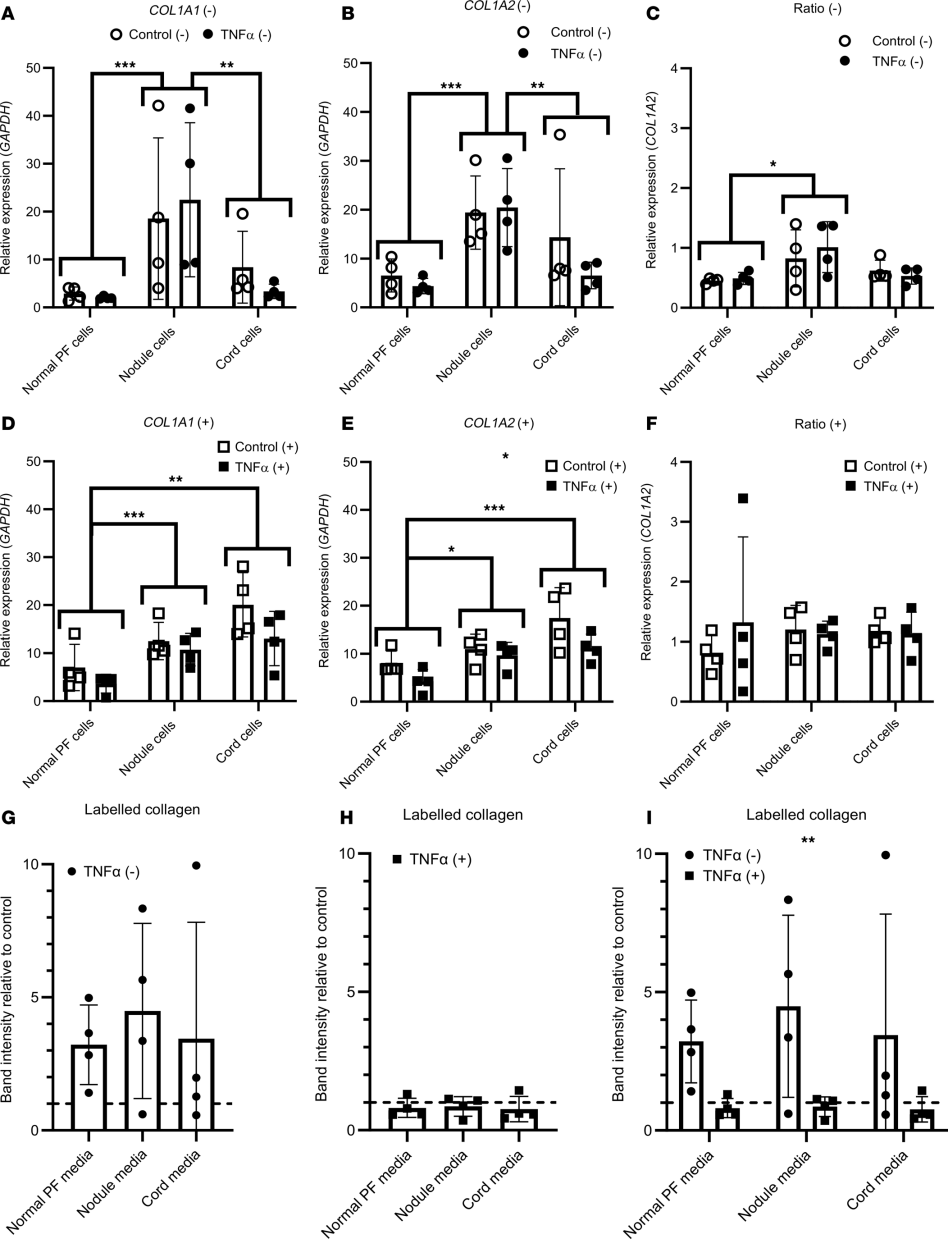
Effect of TNF-α on type I collagen gene expression and collagen protein production in Dupuytren’s cells. (**A**–**F**) Analysis of *COL1A1* (**A** and **D**), *COL1A2* (**B** and **D**), and *COL1A1/COL1A2* (**C** and **F**) gene expression by qPCR in normal PF, Dupuytren’s nodule, and Dupuytren’s cord cells (*n* = 4) after TNF-α treatment in serum-free conditions (–) (**A**–**C**) or in the presence of 10% FCS (+) (**D**–**F**). (**G**–**I**) Densitometric quantification of the relative amounts of radiolabeled collagen present in conditioned media from normal PF, Dupuytren’s nodule, and Dupuytren’s cord cells (*n* = 4) after TNF-α treatment, as compared with control treatments, in serum-free conditions (–) (**G**), the presence of 10% FCS (+) (**H**), or in both conditions (**I**). *P < 0.05, **P < 0.01, and ***P < 0.001 indicated between cell types, or between conditions in the key, by 2-way ANOVA with Holm-Šidák post-hoc test. Data shown in **G** and **H** were tested with 1-way ANOVA and 1-sample t-tests. Sample details are given in Supplemental Table 3. In **E**, the * indicates a significant difference between Control (+) and TNF-α (+). In **I**, the ** indicates a significant difference between TNF-α (–) and TNF-α (+). Please add suitable bracket in the key and move asterisks to indicate the differences.

Previous studies have either treated cells in the presence of serum (27, 28) or have serum-starved cells (30) prior to TNF-α treatment. To determine whether TNF-α increased or inhibited type I collagen synthesis in Dupuytren’s cells, cells derived from Dupuytren’s nodule, cord, or normal PF (Supplemental Table 3) were treated with 10 ng/mL TNF-α in both the presence or absence of 10% serum. Without serum, *COL1A1* and *COL1A2* gene expression was significantly higher in nodule cells than normal PF (*P* < 0.001 for both) and cord cells (*P* = 0.004 and 0.008, respectively) but unaltered by TNF-α treatment (Figure 4, A and B). The *COL1A1/COL1A2* gene expression ratio was higher in cells from nodule than normal PF (*P* = 0.026), but the ratio was consistently less than 2 and unaffected by TNF-α treatment (Figure 4C).

In the presence of serum, *COL1A1* and *COL1A2* gene expression was higher in both nodule (*P* = 0.005 and *P* = 0.015) and cord (*P* < 0.001 for both) cells than in cells from normal PF (Figure 4, D and E). TNF-α treatment significantly lowered *COL1A2* gene expression (*P* = 0.019), but *COL1A1* gene expression was not significantly decreased (*P* = 0.058). The *COL1A1/COL1A2* gene expression ratios were unaffected (Figure 4F).

There were no significant changes in the amount of radiolabeled collagen in the cell culture media following TNF-α treatment in either the absence (Figure 4G) or presence (Figure 4H) of serum as compared with control cultures or between cell types. However, the mean values in TNF-α–treated cultures as compared with controls were all elevated in the absence of serum and decreased in the presence of serum. Comparing the effect of TNF-α treatment in the absence or presence of serum on the relative amount of labeled collagen in cell culture media showed a significant difference between conditions (*P* = 0.001) (Figure 4I).

### Normal PF and Dupuytren’s cells can form 3D tendon–like constructs that fully process type I collagen and assemble aligned collagen fibrils.

**Figure 5:**
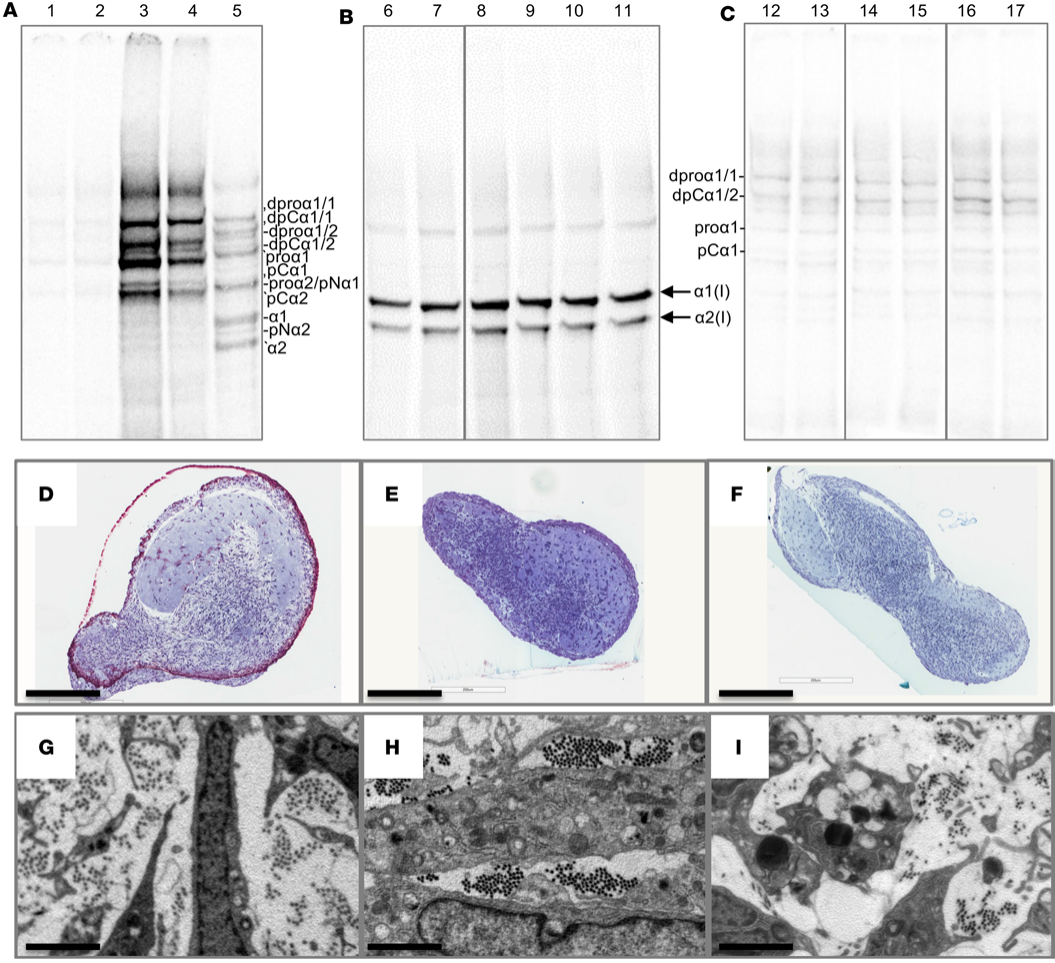
Normal palmar fascia and Dupuytren’s cells fully process procollagen, assemble de novo collagen fibrils, and create tendon-like structures in 3D culture. (**A**–**C**) Representative gel images (1 of 4 replicates) of delayed reduction electrophoresis of the media (**A** and **C**) or salt extractions (**B**) of normal palmar fascia and Dupuytren’s cells grown in 2D (**A**) or 3D (**B** and **C**) culture and labeled with [^14^C]proline. Vertical lines delineate noncontiguous spliced lanes from the same gel (**B**) or different gels (**C**). Fully processed collagen was found in extracellular extracts of the tendon-like constructs (**B**), whereas proforms, and potentially other collagens, were found in the media of both 2D (**A**) and 3D cultures (**C**). The type I procollagen processing intermediates were identified by comparison to labeled standards analyzed under reducing, nonreducing, and delayed reduction conditions and are indicated: “d” indicates disulphide-linked dimers of the respective (/) chains, “pro” indicates procollagen, “pC” indicates pC collagen (lacking the N-propeptide), “pN” indicates pN collagen (lacking the C-propeptide), and “α1” or “α2” indicates the fully processed α chain. For **A**–**C**, lanes 1, 8, and 14 are the control for lanes 2, 9, and 15, which were treated with TNF-α in the absence of serum, and lanes 3, 10, and 16 are the control for lanes 4, 11, and 17, which were treated with TNF-α in the presence of serum. Samples in lanes 6 and 12 were treated with IgG control and in lanes 7 and 13 with anti–TNF-α. Lane 5 is a standard of labeled type I procollagen and processed forms. (**D**–**F**) Semithin toluidine blue–stained sections of 3D tendon–like constructs. Scale bars: 200 μm. (**G**–**I**) Transmission electron microscopy images of 3D tendon–like constructs. Scale bars: 2 μm. Constructs were derived from normal palmar fascia cells (**D** and **G**), Dupuytren’s nodule (**E** and **H**),or Dupuytren’s cord (**F** and **I**) (1 of 4 replicates). Sample numbers are given in Supplemental Table 3.

Cells in 2D culture do not assemble a robust collagenous ECM (31), although they do express type I collagen mRNA (Figure 4, A–F) and synthesize type I collagen trimers. The trimeric type I collagen is primarily produced in the procollagen form and secreted into the cell culture medium where it is partially processed (Figure 5A). The cell layer is often poorly labeled, with procollagen and processed forms being weakly distinguishable above background (not shown).

A fibrin-based 3D culture system was, hence, utilized in which cells replace the fibrin scaffold with a cell-derived collagenous ECM comprising bona fide collagen fibrils and resembling embryonic tendon (32). Human tendon fibroblasts and MSCs have been previously shown to form such structures (33). In the present study, normal PF, Dupuytren’s nodule, and cord cells (Supplemental Table 3) were grown in 3D culture and found to produce tendon-like constructs in 4 weeks. When labeled, these constructs were found to produce fully processed type I collagen (Figure 5B), while labeled procollagen, processing intermediates and potentially other collagens were present in the cell culture media (Figure 5C). The higher molecular weight band in Figure 5B may represent fully processed type III collagen. Ultrastructural analysis of the tendon-like constructs was performed using transmission electron microscopy (TEM) (Figure 5, D–I, and Supplemental Figure 3, A–F). Semithin sections showed the formation of dense cellular structures (Figure 5, D–F, and Supplemental Figure 3, A–C); similar in appearance to tissues formed from fibroblasts and MSCs (34–36). Transmission electron micrographs showed the presence of narrow diameter–aligned collagen fibrils between cells (Figure 5, G–I, and Supplemental Figure 3, D–F) also consistent with previous reports (34–37). Tissues derived from nodular fibroblasts appeared more compact with well-defined fibrils (Figure 5, E and H, and Supplemental Figure 3, B and E).

### TNF-α has limited effects on type I collagen gene expression and does not affect protein production in 3D tendon–like constructs.

**Figure 6:**
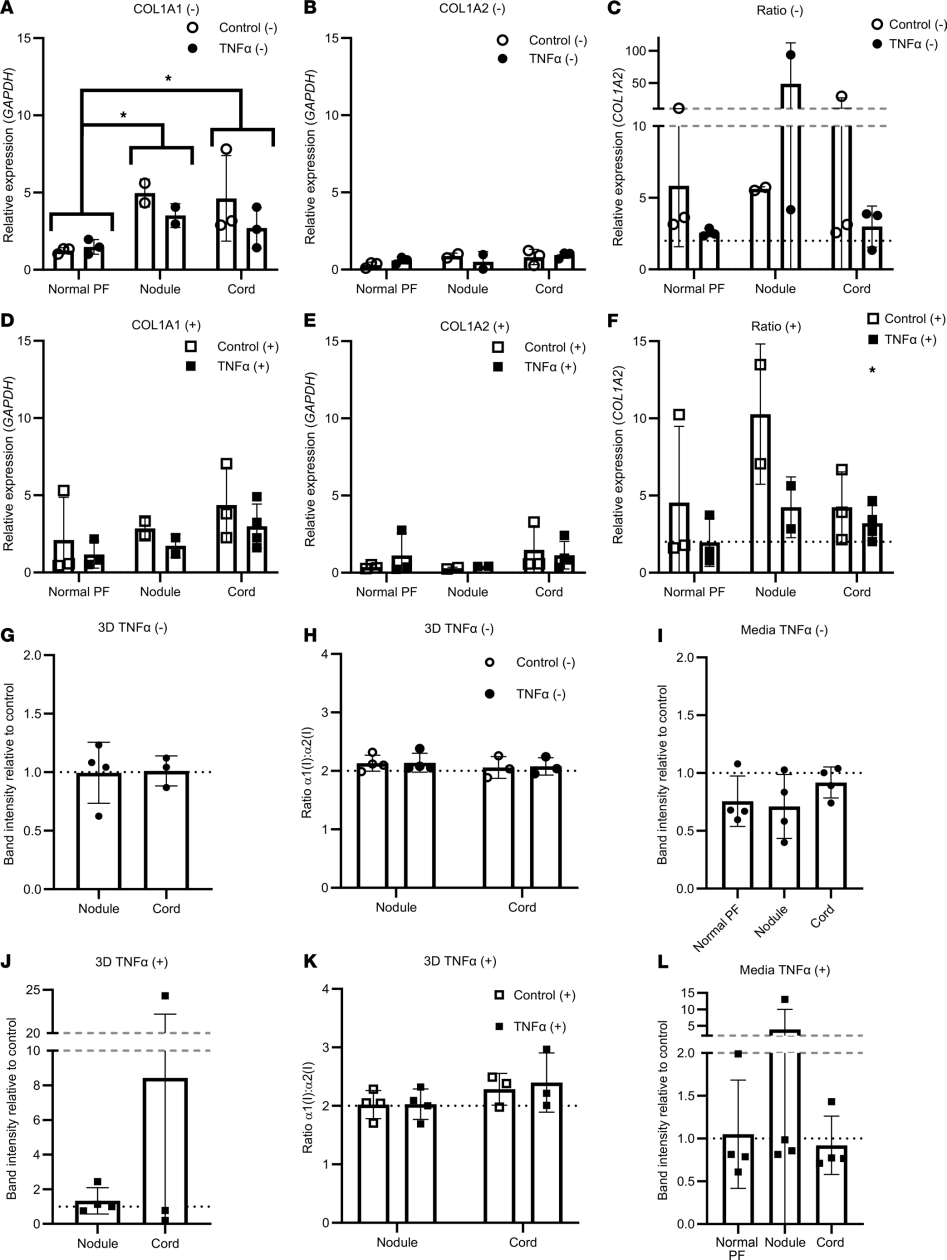
TNF-α has minimal effects on type I collagen synthesis in 3D tendon–like constructs derived from normal PF, Dupuytren’s nodule, and Dupuytren’s cord cells. (**A**–**F**) Analysis of *COL1A1* (**A** and **D**), *COL1A2* (**B** and **E**), and *COL1A1/COL1A2* (**C** and **F**) gene expression by qPCR in 3D tendon–like constructs (after TNF-α treatment in serum free conditions (–) (**A**–**C**), where *n* = 3 for normal PF and cord and *n* = 2 for nodule, or in the presence of 10% FCS (+) (**D**–**F**), where *n* = 3 for normal PF and cord control, *n* = 2 for nodule, and *n* = 4 for cord treated. In **F**, * indicates a significant difference between Control (+) and TNF-α (+). (**G**–**L**) Analysis of type I collagen protein synthesis (**G** and **J**) and type I collagen homotrimer formation (**H** and **K**), where *n* = 4 for nodule and *n* = 3 for cord, and collagen secretion (**I** and **L**), where *n* = 4, by [^14^C]proline labeling in 3D tendon–like constructs after TNF-α treatment in serum free conditions (–) (**G**–**I**) or in the presence of 10% FCS (+) (**J**–**L**). *P < 0.05 by 2-way ANOVA with Holm-Šidák post hoc test, used for **A**–**F**, **H**, and **K**. Data shown in **G** and **J** were analyzed with *t* tests, **I** with 1-way ANOVA, and **L** with 1-way ANOVA on ranks. **G**–**L** were also analyzed with 1-sample *t*/1-sample signed rank tests. Sample details are given in Supplemental Table 3.

Tendon-like constructs derived from normal PF, Dupuytren’s nodule, or cord cells (Supplemental Table 3) were treated with 10 ng/mL TNF-α in the presence or absence of 10% serum and type I collagen gene expression monitored by qPCR (Figure 6, A–F). TNF-α had no effect on *COL1A1* or *COL1A2* gene expression in the presence or absence of serum (Figure 6, A–D), though *COL1A1* gene expression was lower in 3D constructs derived from normal PF cells than those derived from Dupuytren’s nodule or cord cells (*P* = 0.033 and 0.039, respectively) (Figure 6A). There was no significant difference in the *COL1A1/COL1A2* gene expression ratio with TNF-α treatment in the absence of serum (Figure 6C), but the ratio was decreased in the presence of 10% serum (*P* = 0.048) (Figure 6F).

TNF-α had no discernible effect on type I collagen protein synthesis, homotrimer production, or newly synthesized collagens in cell culture media in the absence or presence of serum (Figure 6, G–I). There were no significant differences between cell types or with TNF-α treatment relative to controls for the total α1(I)+α2(I) band intensity (Figure 6, G and J), representing fully processed type I collagen within 3D tendon–like constructs, and there were no significant differences between cell types or with TNF-α treatment relative to controls for collagen secreted into conditioned media (Figure 6, I and L). Unlike for 2D cultures, TNF-α did not have opposing effects on labeled collagens present in media in the absence or presence of serum (Supplemental Figure 4) The α1(I)/α2(I) ratio for fully processed type I collagen extracted from 3D tendon–like constructs was not significantly different between conditions or cell types and was not significantly greater than 2 (Figure 6, I and L), indicating that 3D tendon–like constructs primarily synthesized heterotrimeric rather than homotrimeric type I collagen.

### Anti-TNF did not reduce type I collagen synthesis in 3D tendon–like constructs or type I collagen homotrimer synthesis in Dupuytren’s tissue.

**Figure 7:**
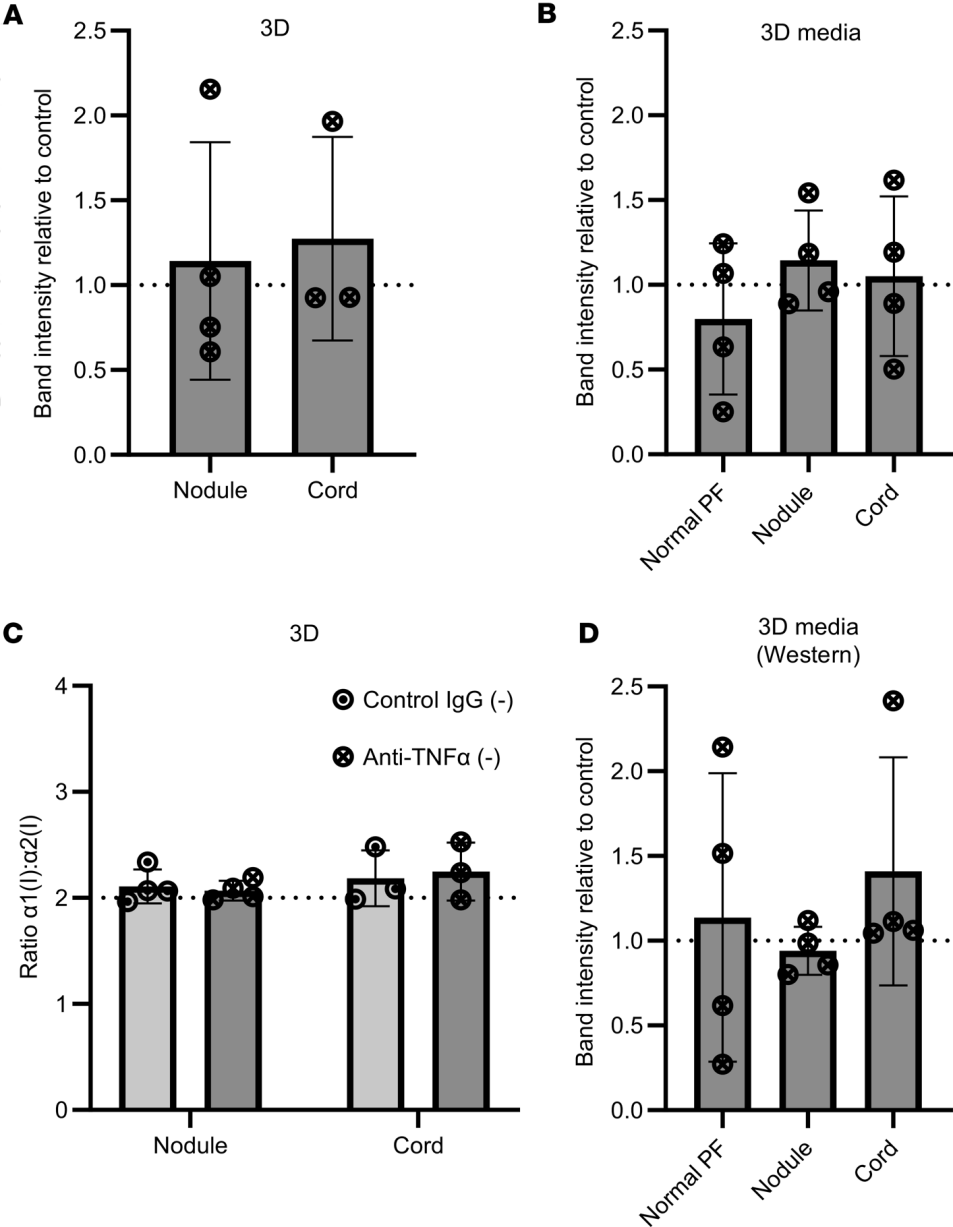
Anti–TNF-α does not reduce type I collagen synthesis in fully contracted 3D tendon–like constructs. (**A**–**D**) Analysis of type I collagen protein synthesis (**A**), collagen secretion (**B**), and type I collagen homotrimer formation (**C**) by [^14^C]proline labeling in 3D tendon–like constructs, and type I collagen secretion by Western blotting (**D**), after control IgG or anti–TNF-α treatment in serum free conditions (–). *n* = 4 except for **A** and **C**, where *n* = 3 for cord. Sample details are given in Supplemental Table 3. Data shown in **A** were analyzed with a *t* test, in **B** and **D** by 1-way ANOVA, and in **C** by 2-way ANOVA. All data was also analyzed with 1-sample *t*/1-sample signed rank tests.

It may be the case that 3D tendon–like constructs already contain active TNF-α. Hence inhibition rather than addition of TNF-α was next tested. To determine if anti–TNF-α would reduce type I collagen synthesis or processing in 3D tendon–like constructs, the antibody was added to fully formed constructs in the absence of serum. Anti–TNF-α had no effect on the total α1(I)+α2(I) band intensity, representing fully processed type I collagen within and synthesized by 3D tendon–like constructs, as compared with IgG controls (Figure 7A). The newly synthesized collagens in cell culture media were similarly unaffected by anti–TNF-α treatment, as compared with IgG controls, and there were no significant differences between cell types (Figure 7B). As observed for TNF-α treatment and controls (Figure 6, H and K) the α1(I)/α2(I) ratio for fully processed type I collagen extracted from 3D tendon–like constructs was not significantly greater than 2, did not change with anti–TNF-α treatment, and was not different between cell types (Figure 7C).

Due to the presence of type I collagen proforms in cell culture media, it was not possible to unequivocally distinguish between type I collagen and other radiolabeled secreted collagens such as type III. Western blotting using an antibody to the triple helical domain of the α1(I) chain was therefore used to determine if anti–TNF-α affected type I collagen protein secretion from constructs. Again, no differences in the amount of type I collagen in media from tendon-like constructs were detected with anti–TNF-α treatment (Figure 7D).

**Figure 8:**
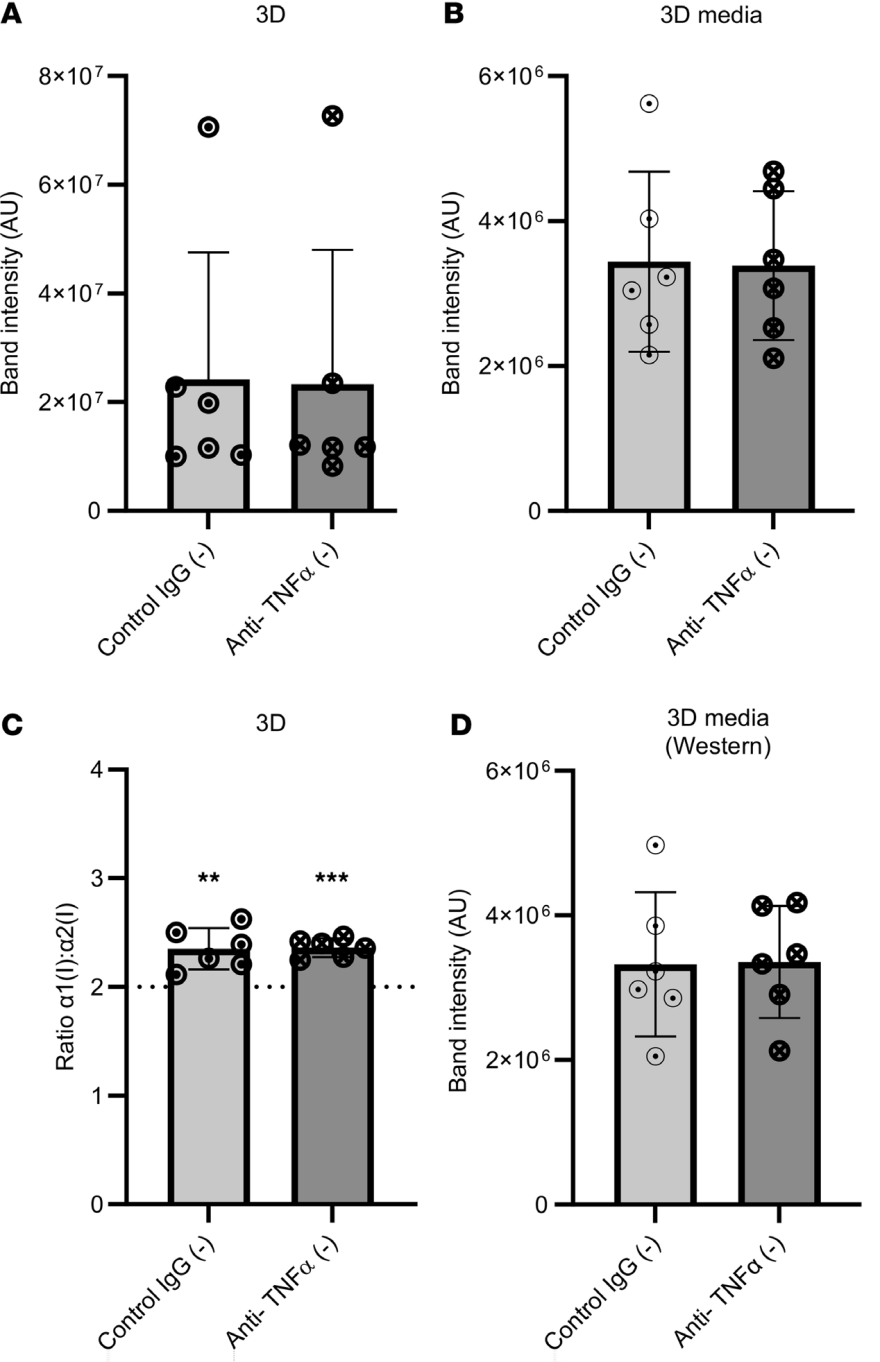
Anti–TNF-α does not reduce type I collagen synthesis in precontracted 3D tendon–like constructs. (**A**–**D**) Analysis of type I collagen protein synthesis (**A**), collagen secretion (**B**), and type I collagen homotrimer formation (**C**) by [^14^C]proline labeling in 3D tendon–like constructs, and type I collagen secretion by Western blotting (**D**), after control IgG or anti–TNF-α treatment (*n* = 6) in serum free conditions (–). Sample details are given in Supplemental Table 3. **P < 0.01 and ***P < 0.001 by 1-sample *t* tests against a reference value of 2. All data was also analyzed using paired *t* tests.

Since anti-TNF has shown promise in early stage Dupuytren’s disease, we tested the effect of anti-TNF treatment on collagen synthesis during formation of constructs derived from Dupuytren’s cells (Figure 8). Again anti–TNF-α had no effect on the total α1(I)+α2(I) band intensity as compared with IgG control (Figure 8A), on the collagens in media (Figure 8B), or on the α1(I)/α2(I) ratio (Figure 8C). Western blotting confirmed there were no differences in the amount of type I collagen in media from tendon-like constructs due to anti–TNF-α treatment (Figure 8D). Interestingly, the α1(I)/α2(I) ratio in these precontracted constructs was greater than 2, though it was unaffected by anti–TNF-α treatment (Figure 8C).

**Figure 9:**
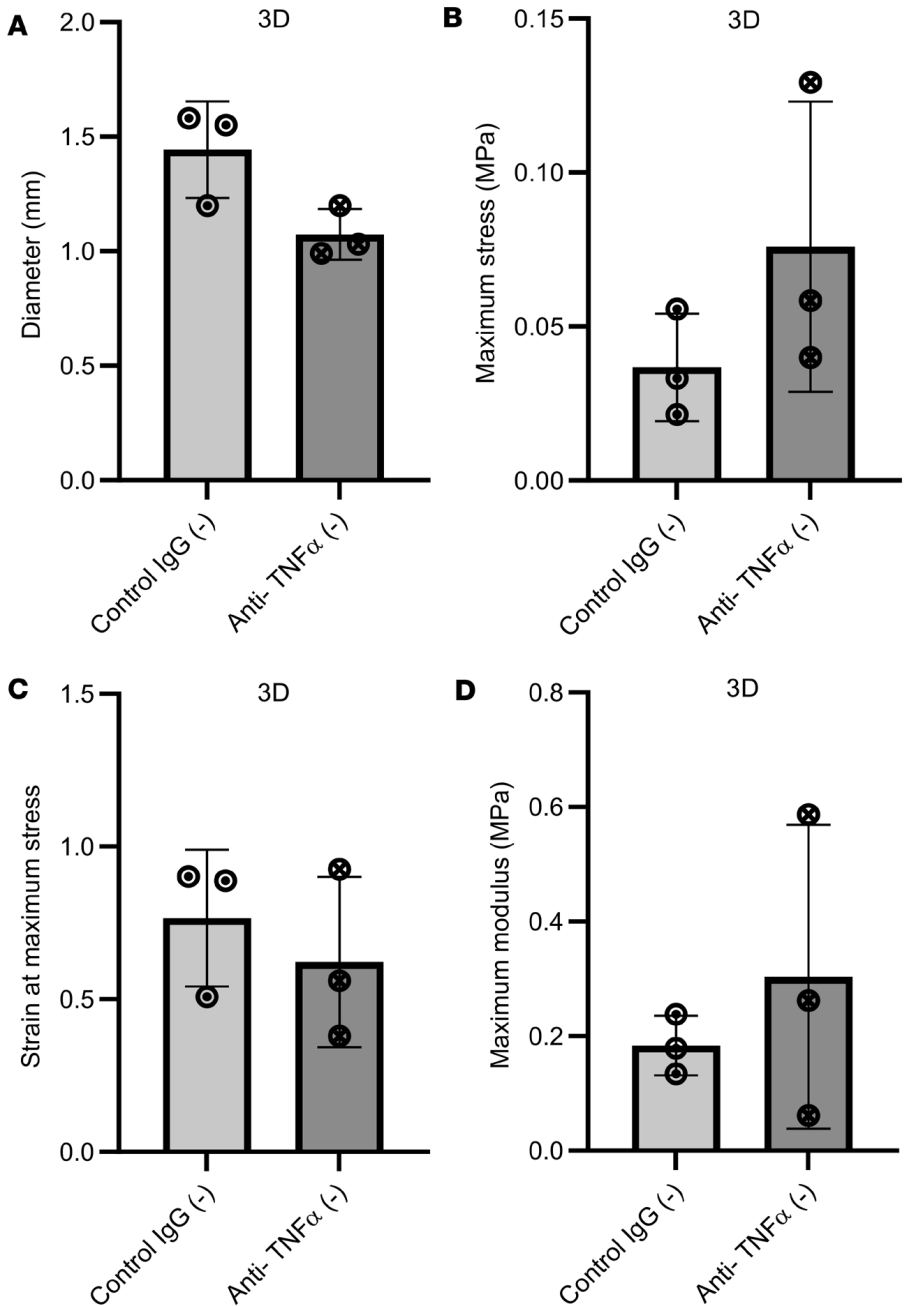
Anti–TNF-α does affect tensile mechanical properties in 3D tendon–like constructs. (**A**–**D**) Analysis of diameter (**A**), maximum stress (**B**), strain at maximum stress (**C**), and maximum modulus (**D**) after control IgG or anti–TNF-α treatment (*n* = 3) in serum-free conditions. Sample details are given in Supplemental Table 3. No significant differences were detected using paired *t* tests.

Given that anti-TNF has been previously shown to influence cell contractility (24) and that contractility can influence ECM stiffness (38), we evaluated whether anti-TNF could influence tissue stiffness in 3D constructs. However, treatment with anti–TNF-α had no effect on the intrinsic tensile mechanical properties (Figure 9), including modulus, which provides a size-corrected measure of stiffness.

**Figure 10:**
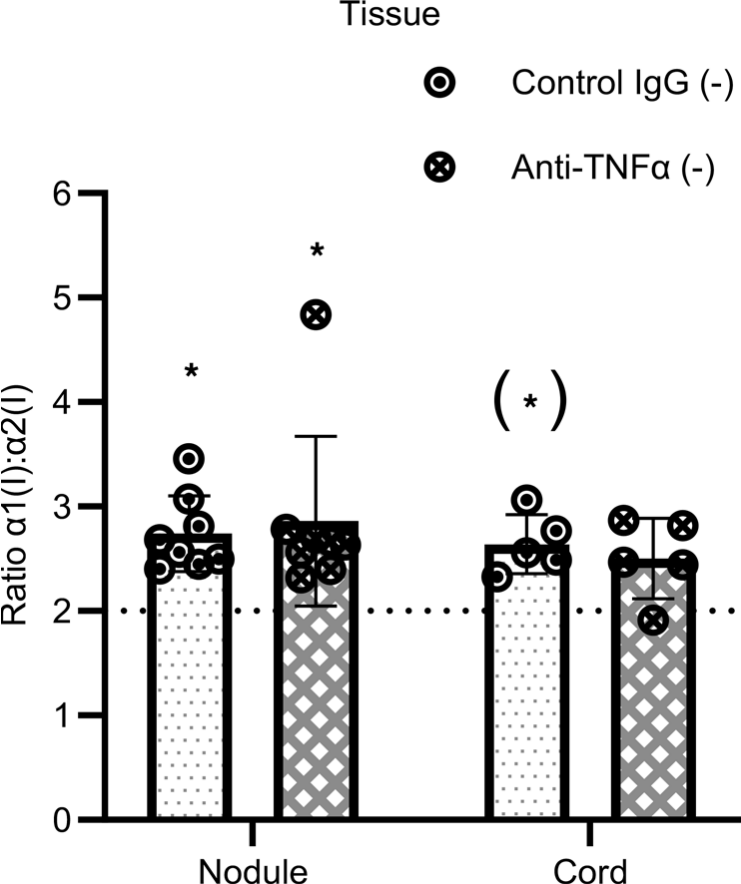
Anti–TNF-α does not prevent type I collagen homotrimer formation in Dupuytren’s tissue explants. Analysis of type I collagen homotrimer formation by [^14^C]proline labeling in Dupuytren’s nodule (*n* = 8) or cord (*n* = 5) tissue explants after treatment with control IgG or with anti–TNF-α in serum free conditions (–). *P < 0.05 (1-sample *t* tests against a reference value of 2), (*) indicates significance only in a 1-tailed 1-sample *t* test. Data were also analyzed by 2-way ANOVA.

Unlike fully contracted 3D tendon–like constructs, Dupuytren’s tissue explants were found to synthesize homotrimeric type I collagen (Figure 1, E and F). Nodule and cord tissue explants from patients with Dupuytren’s (Supplemental Table 3) were therefore treated with control IgG or anti–TNF-α to determine if anti-TNF would reduce active synthesis of type I collagen homotrimer. For these experiments, the total or secreted collagens were not compared due to variability in the size of tissue explants between conditions. The α1(I)/α2(I) ratio was significantly greater than 2 for nodule explants (*P* = 0.010 and 0.008 for control and treatment, respectively), indicative of type I collagen homotrimer synthesis (Figure 10). The cord control appeared significantly greater than 2 only when using a 1-tailed 1-sample *t* test (*P* = 0.043). There were no significant differences between anti–TNF-α and control IgG treatment, indicating that anti–TNF-α did not reduce active synthesis of type I collagen homotrimer.

## Discussion

In this study, we showed increased *COL1A1* and *COL1A2* mRNA as well as an increased *COL1A1/COL1A2* mRNA ratio in Dupuytren’s tissue as compared with normal PF (Figure 1, A–C). The results presented are in accordance with previous studies that showed increased *COL1A1* mRNA in Dupuytren’s tissue compared with an unaffected transverse ligament of the palmar aponeurosis (39) and increased *COL1A1* but not *COL1A2* mRNA in Dupuytren’s tissue, as compared with shoulder capsule (40). For surgical waste human tissue samples, we did not distinguish between nodule and cord for RNA analysis, although a prior study found both *COL1A1* and *COL1A2* mRNA was higher in nodule as compared with cord, despite lower type I collagen protein being present in nodule tissue (41).

One previous study indicated the presence of type I collagen homotrimer in Dupuytren’s tissue, with an increase from 5% in normal PF to 9% in Dupuytren’s, utilizing pooled samples (21). Utilizing metabolic labeling with [^14^C]proline, we found a consistent increase in the α1(I)/α2(I) polypeptide chain ratio for newly synthesized collagen in Dupuytren’s tissue samples, but we noted a skewed distribution with some samples showing a particularly high α1(I)/α2(I) chain ratio (Figure 1, E and F). We hypothesized that individual variation or samples analyzed at different stages of disease progression may be responsible for the relative proportion of newly synthesized collagen; however, no association with demographics data or disease stage was observed (Figure 2). It is possible that the current study is underpowered to accurately identify factors or interactions that may direct high type I collagen homotrimer synthesis or that the granularity of the demographics data is insufficient. The mean percentage of homotrimeric collagen is higher than that reported by Ehrlich et al., although there is a cluster of several samples apparently synthesizing type I collagen homotrimer to a similar extent, at around 10% (21). Notably our study also provides a snapshot of type I collagen homotrimer synthesis, whereas Ehrlich et al. analyzed accumulated homotrimeric type I collagen. Interestingly, precontracted, but not fully contracted, tendon-like constructs appeared to synthesize type I collagen homotrimer, as the α1(I)/α2(I) chain ratio was significantly greater than 2. Hence, it may be the case that active cell contractility is related to homotrimer synthesis.

We noted an absence of detectable type I collagen protein synthesis in normal PF by radiolabeling (Figure 1D), obtaining similar results for healthy canine CCL and postnatal equine SDFT. These findings indicate that the type I collagen synthesis detected in Dupuytren’s and ruptured CCL explants is not due to an acute injury response to dissection. In ruptured canine CCL, expression of *COL1A1* and *COL1A2* mRNA was increased (Figure 3, A and B). A previous study in canine CCL indicated a significant increase in *COL1A2* mRNA but not of *COL1A1* in ruptured ligaments (42), while *COL1A1* mRNA was found to be increased in ruptured human anterior cruciate ligament by in situ hybridization (43). In the present study, the *COL1A1/COL1A2* ratio was significantly higher in ruptured canine CCLs, but the ratio did not exceed 2, and type I collagen homotrimer synthesis was not apparent (Figure 3E). Gene expression of *COL1A1* and *COL1A2* in equine SDFT reduced dramatically after 2 years of age (Figure 3G), and type I collagen protein synthesis was only detected in fetal tendon (Figure 3F), consistent with the known high-level type I collagen expression during embryonic tendon development (44). No age-related changes in the *COL1A1/COL1A2* mRNA ratio were observed in equine SDFT.

Despite a lack of type I collagen protein synthesis, we noted labeled bands in normal PF (Figure 3A), canine CCL (Figure 3E), and SDFT (Figure 3F) tissue extracts. Attempts to identify the labeled proteins using [^13^C] proline labeling, band excision and mass spectrometry, or by Western blotting to candidate collagens were unfortunately not informative. However, given the incorporation of [^14^C]proline, we expect that these proteins are collagenous in nature or are proline rich.

Interestingly we noted little correlation between the *COL1A1/COL1A2* mRNA ratio and the α1(I)/α2(I) chain ratio in Dupuytren’s tissue (Figure 1G). In addition, the increased *COL1A1/COL1A2* mRNA ratio in Dupuytren’s tissue as compared with normal PF (Figure 1C) was less significant than the increased α1(I)/α2(I) chain ratio in Dupuytren’s samples (Figure 1E), while canine CCLs generally had more *COL1A2* than *COL1A1* mRNA (Figure 3D). Early reports indicated that the *COL1A1/COL1A2* mRNA ratio is maintained at 2:1 (45). However, there appears to be buffering in the system as the 2:1 polypeptide chain ratio is maintained when the mRNA ratio is disrupted to 1:1 by a nonfunctional *COL1A1* allele (46) and overexpression of both genes results in 2:1 heterotrimers, unless *COL1A1* is in large excess (47). Cytosolic mRNA binding proteins have been reported to coordinate translation of *COL1* mRNAs (48), and specific type I collagen C-propeptide sequences favor its heterotrimerization (49, 50). Hence, the mRNA ratio cannot provide a direct readout of the extent of homotrimerization.

While we did not detect a difference in *COL1A1* gene expression in 2D cell cultures with TNF-α treatment in the presence or absence of serum, *COL1A2* gene expression was reduced by TNF-α in the presence of serum (Figure 4) in accordance with a prior study (29). TNF-α had opposing effects on new collagen protein secretion in the presence or absence of serum with a more repressive effect in 10% serum. This may relate to the presence of TGF-β in serum, antagonistic TNF-α, and TGF-β signaling and the inhibitory effect of serum α2-macroglobulin on cytokine signaling (29). TGF-β is estimated to be present in 10% serum at approximately 10 ng/mL (51), which is comparable with that used for exogenous treatment (29, 30). Serum-containing media, therefore, contain sufficient TGF-β to drive *COL1A2* gene expression, which can be at least partially counteracted by equimolar TNF-α. Prior work indicated that TNF-α increased *COL1A1* mRNA in palmar fibroblasts, from patients with Dupuytren’s though not in Dupuytren’s nonpalmar dermal fibroblasts or in palmar fibroblasts from those unaffected by Dupuytren’s, though it is unclear whether treatments were carried out in the presence of serum.

Adult human tenocytes have been previously found to form 3D tendon–like constructs when seeded into a fibrin gel scaffold (33), although these were younger (29 ± 7.5 years) than the normal PF and Dupuytren’s cells used in our study (range, 57–76 years). Therefore, cells derived from normal PF retain the capacity to synthesize a new collagenous ECM even up to 69 years of age (Supplemental Table 3). Prior radiolabeling of newly synthesized collagen in 3D tendon–like constructs detected proforms as well as fully processed collagen (52) following 1 hour of labeling. In the present study, fully processed collagen was primarily detected within constructs, which can be attributed to a 3-hour chase, following overnight labeling, allowing the majority of the newly synthesized labeled collagen to traverse the secretory pathway and be fully proteolytically processed to tropocollagen. In contrast the media contained primarily proforms, likely due to dilution of the procollagen substrate and processing enzymes in the liquid environment, and potentially due to other collagens.

In 3D tendon–like constructs the *COL1A1*/*COL1A2* ratio was decreased with TNF-α treatment in the presence of serum (Figure 6), indicating potential differential effects on *COL1A1* and *COL1A2* gene expression in 3D culture. However, this was not reflected in an altered type I collagen α1(I)/α2(I) protein chain ratio. Unlike in 2D culture, no differential effects of TNF-α on collagen protein production were observed in 3D culture, either for secreted protein in the media or that within the constructs. These findings may reflect residual serum proteins being retained within the fully formed 3D tendon–like constructs, differences in signaling responses in 3D culture, or the presence of a collagenous ECM barrier that could prevent TNF-α from interacting with cells.

In the present study, anti-TNF was not able to reduce type I collagen synthesis in fully contracted or precontracted 3D tendon–like constructs or their media, nor was it able to modulate type I collagen homotrimer synthesis in Dupuytren’s tissue (Figure 7, Figure 8, Figure 9). This appears in contrast to the reported effect of anti-TNF on *COL1A1* gene expression in 2D Dupuytren’s myofibroblasts (24) and reduced type I procollagen in patient nodules 2 weeks after anti-TNF (adalimumab) injection (25). Although we did not test anti-TNF in 2D culture, tests in 3D culture or tissue explants are more relevant to the in vivo condition and Nanchahal et al. similarly did not detect reduced *COL1A1* expression in injected nodules. Interestingly, in vivo type I procollagen (measured with an ELISA for the N-propeptide, as a procollagen proxy) was only reduced with the highest anti-TNF dose (*P* = 0.019), which became available during the dose-ranging study (25), whereas total type I collagen was not reduced with anti-TNF treatment, which is not unexpected given the relative timescales of treatment and prior disease progression.

Herein, we utilized IgG as a control for the anti-TNF treatments, whereas saline injection was used as control for the phase 2a trial (25), and the control for prior 2D culture studies is ambiguous (24). The anti-TNF biologic infliximab does not bind soluble or membrane-bound mouse TNF-α, but it improves mobility and suppresses joint inflammation, weight loss, and pain in mice overexpressing mouse TNF-α (TNF^DARE^) (53). Indeed, Fc receptor–mediated effects contribute to the therapeutic response to infliximab and adalimumab in mouse models of inflammatory bowel disease (IBD) (54), and patients with IBD only respond to full IgG1 anti-TNF therapies via macrophage IL-10 signaling (55–57). Indeed immune cell populations including macrophages are present in Dupuytren’s disease tissue (24, 58). Hence, it is possible that the beneficial effects of the anti-TNF adulimumab in Dupuytren’s disease could be Fc mediated and not related to modulation of collagen synthesis. Overall, we conclude that modulation of TNF-α signaling in Dupuytren’s cells or tissue does not have a direct effect on collagen production. It may be, however, that anti-TNF has beneficial effects in dampening inflammation in nodules, or on myofibroblast activation or contractility, in early-stage Dupuytren’s disease. Hence antifibrotic effects of anti-TNF may be indirect, rather than targeting type I collagen synthesis per se.

## Methods

### Sex as a biological variable.

Our study included both male and female human donors. Gender was captured in patient-reported demographics and is provided in Supplemental Tables 1–3. Gender was not associated with greater type I collagen homotrimer synthesis (Figure 2). The sex of all animal samples was not available.

### Sample collection.

Excised Dupuytren’s tissue (fibrotic PF) and control nondiseased normal PF were taken from patients undergoing surgery for Dupuytren’s contracture and carpal tunnel syndrome, respectively. Surgery was carried out within Royal Liverpool and Broadgreen University Hospitals NHS Trust (now Liverpool University Hospitals NHS Foundation Trust) or the Warrington and Halton Hospitals NHS Foundation Trust (now Warrington and Halton Teaching Hospitals NHS Foundation Trust). Self-reported demographics were collected by questionnaire, and for the patients with Dupuytren’s, disease stage information was collected from clinical records. Equine SDFT and healthy canine CCL were collected as previously described (59, 60). Ruptured CCL, otherwise discarded as clinical waste, was obtained from dogs undergoing stifle/knee stabilization at the University of Liverpool’s Small Animal Teaching Hospital. Surgical samples were reserved in cold sterile saline and processed on the day of collection according to the selected analysis method. During processing of Dupuytren’s samples, perinodular fat was first removed. Samples were only separated into cord and nodule where possible and where indicated.

### Materials.

All reagents were obtained from Sigma-Aldrich unless otherwise stated.

### Tissue pulse-chase with ^14^C-L-proline.

Tissue samples were aseptically dissected into pieces, with an estimated wet weight of 25–50 mg or volume of ~25–50 μL each, under PBS or DMEM (both from Thermo Fisher Scientific), containing penicillin/streptomycin (Thermo Fisher Scientific) (1% v/v). After a 30- to 60-minute preequilibration at 37°C in DMEM containing penicillin/streptomycin (1% v/v), L-glutamine (2 mM; Thermo Fisher Scientific), L-ascorbic acid 2-phosphate (200 μM), and β-aminopropionitrile (400 μM) (labeling media) with ~1–5 pieces per 1 mL of media, pulse-chase experiments were performed in labeling media supplemented with 2.5 μCi/mL [^14^C]proline (PerkinElmer). After incubation in supplemented labeling media for 18 hours, tissue samples were transferred to labeling media without [^14^C]proline for 3 hours. Subsequently, 1–2 tissue pieces were extracted in 100 μL aliquots of salt extraction buffer (1M NaCl, 25 mM EDTA (Thermo Fisher Scientific), 50 mM Tris-HCl [pH 7.4]) containing protease inhibitors. Extracts or media aliquots were analyzed by electrophoresis on 6% Tris-Glycine gels (Thermo Fisher Scientific) with delayed reduction (61) or on 4% Tris-Glycine gels (Thermo Fisher Scientific) under reducing conditions. The gels were fixed in 10% methanol and 10% acetic acid, dried under vacuum, and exposed to a phosphorimaging plate (BAS-IP MS). Phosphorimaging plates were processed using a phosphorimager (Typhoon FLA7000 IP, GE Healthcare/Cytiva) and densitometry carried out using ImageQuant (GE Healthcare/Cytiva). The α1(I)/α2(I) chain ratio was corrected for the relative amount of proline residues in each chain [240 in α1(I) and 204 in α2(I) such that the ratio was divided by 1.1765] before analysis, and converted to a percentage homotrimeric collagen using the formula: (ratio –2) × 100/ratio.

### Cell isolation and culture.

Tissue samples were dissected into small pieces and digested with –2 mg/mL (275 U/mL) collagenase type II (Worthington) in 30 mL DMEM containing 1 % (v/v) penicillin/streptomycin and 2% FBS (F7524, Batch 024M3398, Sigma-Aldrich), at 37°C overnight in a rotary shaker. Dupuytren’s cell suspensions were strained in a 70 μm cell strainer, centrifuged for 10 minutes at 1,000*g* and the cell pellets resuspended in DMEM with L-glutamine supplemented with 10% FBS, 1 % (v/v) penicillin/streptomycin, and 0.5 μg/mL fungizone (Thermo Fisher Scientific). Cells were seeded at 0.5 × 10^5^ to 3.0 × 10^5^ cells per cm^2^ in a T25 or T75 flask, grown at 37°C in 5% CO_2_ (BOC gases) and cryopreserved at passage 0 or 1. Normal PF samples were digested with 2 mg/mL collagenase in 15 mL of media with cell straining omitted for 3 of the 4 samples. Cells were seeded directly into 1 well of a 6-well plate and cryopreserved at passage 1.

Dupuytren’s cells split 1:2 at passage 1, 2, or 3, and normal PF cells at passage 3 or 4 were used to establish 3D tendon–like constructs as described (34), with the following modifications. Constructs for ultrastructural analysis and radiolabeling were established in 24-well rather than 6-well plates as were those for qPCR, excepting those derived from normal PF cells and cord-derived cells from sample 161012-62-F (Supplemental Table 3). To form constructs in 24-well plates (Supplemental Figure 5), pins were positioned 5 mm apart, the volume of thrombin to use was titrated in advance for each batch, and constructs were seeded with 2.5 × 10^5^ cells per well. For 6-well plates, constructs were seeded with 6 × 10^5^ cells per well. Scoring and media changes took place 3 times a week (every 2–3 days).

### Cell and tissue treatments.

Prior to treatment, the media were changed to that supplemented with nonessential amino acids for 2D cell cultures, L-ascorbic acid 2-phosphate (200 μM) and β-aminopropionitrile (400 μM), without serum as indicated or for explants, and cultures were incubated at 37°C in 5% CO_2_ for 16–18 hours. The media were then replaced with that containing 10 ng/mL human TNF-α (300-01A, Peprotech), vehicle control, mouse anti–human TNF-α antibody (MAB610, clone 28401, R&D Systems, ) or mouse IgG1 Isotype Control (MAB002, 11711, R&D Systems), along with 2.5 μCi/mL [^14^C]proline overnight as required. Labeling media were reserved and chase media without supplements were added for 3 hours. Cells were used at passage 3 or 4, and at 80% confluence, constructs were fully contracted and tissue samples were treated on the day of collection. Constructs were also treated with TNF-α antibody or IgG control and radiolabeled overnight, 16 days prior to full contraction (12 days after initiation). Unlabeled constructs were stored in PBS-soaked gauze at –20°C for biomechanical testing. Labeled tissues and 3D constructs were snap frozen and labeled collagen was extracted with salt-extraction buffer. Salt extracts and labeling media were analyzed by delayed reduction electrophoresis as described above. For media, all labeled collagens and proforms were used for quantification and denoted as collagen.

### qPCR.

Tissue samples and 3D tendon–like constructs were immersed in RNAlater (Qiagen) and stored at –20°C until analysis. The tissue was homogenized with a Mikro-Dismembrator (Sartorius) or a pestle and mortar in 1 mL of TRI Reagent (Sigma-Aldrich). In total, 0.1 mL 1-bromo-3-chloropropane (BCP) (Sigma-Aldrich) was added to the homogenate and centrifuged for 15 minutes at 12,000*g* at 4°C. Then, 1 μg of total RNA was reverse transcribed with M-MLV reverse transcriptase using random primers according to the manufacturer’s protocol (Promega). qPCR was performed in a 25 μL reaction volume containing cDNA (5 ng), primers designed for the gene of interest (Eurogentec; Table 1), and GoTaq(R) qPCR Master Mix (Promega). Samples were run on an AB 7300 Real Time PCR System (Applied Biosystems) using the following amplification conditions: 2 minutes at 95°C followed by 40 cycles of 15 seconds at 95°C and 1 minute at 60°C. Gene expression was calculated relative to GAPDH, which was determined to be a suitable reference gene after assessing its stability using the geNorm method (62).

RNA was first extracted from cultured cells using Trizol (Thermo Fisher Scientific). An equal volume of 100% ethanol was added to the aqueous phase after phase separation with chloroform and then loaded onto an RNeasy column (Qiagen). RNA was purified following the manufacturer’s protocol with the following modifications: 30-second rather than 15-second spins, only 1 wash with buffer RPE, an optional spin to exclude RPE buffer carryover, and the final elution carried out 1–2 minutes after adding RNase-free water. Constructs were homogenized using a steel ball lysing matrix and a FastPrep 24 tissue homogenizer (MP Biomedicals). RNA was extracted from homogenized samples using a RNeasy kit (Qiagen) as per the manufacturer’s instructions. cDNA was synthesized in a 25 μL reaction from 0.5–1 μg total RNA for cells and 0.15 μg for constructs as described. cDNA synthesis and qPCR were performed as described (63) using human primers shown in Table 1.

### TEM.

TEM was carried out as described (37) but modified for constructs derived from normal PF cells, for which an initial fixation (~5 minutes) was carried out in 4% paraformaldehyde in PBS before transferring half the fixed construct to 2.5% glutaraldehyde (Thermo Fisher Scientific) in 0.1M cacodylate. Semithin (0.5 μm) sections were stained with 1% toluidine blue and visualized by light microscopy.

### Western blotting.

Equal volumes of media were analyzed by electrophoresis on 6% Tris-Glycine gels (Thermo Fisher Scientific) with delayed reduction (61) and proteins transferred to nitrocellulose membrane. Membranes were blocked overnight in 5% nonfat dried milk powder in PBS with 1% Tween 20 (PBS-T). Washes were carried out once for 15 minutes and 3 times for 5 minutes with PBS-T before and after each antibody incubation. The primary antibody was anti–collagen α1(I) (ab138492, clone EPR7785, Abcam) at 1:1,000 and the secondary mouse anti–rabbit IgG-HRP (sc-2357, Santa Cruz Biotechnology) at 1:10,000. Chemifluorescence detection was performed using Pierce ECL Plus Western Blotting Substrate (Thermo Fisher Scientific) and a Typhoon FLA7000 IP imager with Cy2 filter. Densitometry was carried out using ImageQuant (GE Healthcare/Cytiva). The anti–collagen α1(I) recognizes the pepsinized mature form of type I collagen (64); hence, the epitope resides in the triple helical domain.

### Biomechanical testing.

Samples were defrosted on the day of testing, mounted with tape on acetate window frames (window sides cut prior to tensile testing), and submerged in PBS until testing. Average diameters were derived from measurements taken at 3 points along the sample length using a graticule scale and optical microscope (Nikon Diaphot). Tensile testing was conducted using a Universal Testing System (Instron 68SC-5, Norwood) and 5N load cell (Model No. 2530-5N). Tendons were kept hydrated between spring-loaded grips, to apply a consistent compressive force to hold all samples, with a nonheated humidifier (Ultrasonic Wave). Constructs underwent 4 cycles of preconditioning between 0N and 0.01N, with a 90-second recovery period between each cycle followed by a ramp to 0.01N before holding the strain for 60 seconds to allow the tissue stress to relax. All loading/unloading cycles and prerelaxation ramps were carried out at a strain rate of 20%/min. Following stress relaxation, the load was reduced to 0N, and the samples were allowed to recover for a further 90 seconds before a final ramp to failure at 20%/min. Sample dimensions, applied force, extension, and test time were used to calculate maximum stress, strain at maximum stress, and the maximum tangent modulus, using Excel (Microsoft).

### Statistics.

Data analysis was performed using SigmaPlot Version 14.0 (Systat), and graphing was carried out with GraphPad Prism 8 or 9 for Windows (GraphPad Software), unless otherwise indicated. Plots show individual data points together with the mean ± 1 SD unless otherwise indicated. Following a log_10_ transformation as required, COL1 gene expression data and sample ages were analyzed using a 2-tailed Welch’s *t* test. Polypeptide chain ratios were transformed with an inverse function and analyzed using a 1-sample 1- or 2-tailed *t* test (2-tailed unless stated otherwise). qPCR and collagen synthesis data were analyzed by 1-way ANOVA for 1 factor, by 2-way ANOVA with 2 factors, by a 2-tailed paired *t* test for 2 conditions, or with a 1-sample *t* test for comparison to a single value. A prior suitable Box-Cox transformation was carried out if assumptions of normality and equal variance were not met. If no suitable transformation was found, an equivalent nonparametric test — e.g., Kruskal-Wallis 1-way ANOVA on ranks or a 1-sample signed rank test — was used. A *P* value less than 0.05 was considered significant. PCA and associated graphing was carried out using Minitab Statistical Software Version 18 as was Box-Cox data transformation. For PCA incorporating demographic data, self-reported textual data were coded in a binary manner except for diabetes, where diet-controlled diabetes (coded 1) was distinguished from type 1 diabetes (coded 2); smoking, where previous smokers were coded 1 and current smokers coded 2; alcohol consumption, where those consuming below the recommended limit were coded 1 and those above coded 2; and exercise, which was coded from 0 to 3 based on apparent intensity/duration.

### Study approval.

Human samples were collected with NHS Health Research Authority (HRA) and Regional Research Ethics Committee (GM East REC) approval (REC13/NW/0352 and REC 14/NW/0162) and with full informed written patient consent. Animal samples were collected with approval from the University of Liverpool Veterinary Research Ethics Committee (VREC62, VREC63, VREC186, and RETH00000553).

### Data availability.

Data values presented in the manuscript are provided in the Supporting Data Values file.

## Author contributions

Conceptualization was contributed by EGCL. Funding acquisition was contributed by EGCL. Resources were contributed by G Cheung, DB, RP, EJC, and PDC. Methodology was contributed by KW, KJL, and EGCL. Investigation was contributed by KW, KJL, ELB, AC, JAG, G Cooper, BG, and LAB. Data curation was contributed by KW, KJL, ELB, and EGCL. Project administration was contributed by RP, EJC, PDC, and EGCL. Supervision was contributed by EJC, PDC, and EGCL. Formal analysis was contributed by KW, KJL, ELB, NSOY, LAM, BG, LAB, and EGCL. Visualization was contributed by KW, KJL, ELB, and EGCL. Writing of the original draft was contributed by EGCL. Review and editing of the manuscript were contributed by KW, KJL, ELB, JAG, G Cooper, NSOY, LAM, G Cheung, DB, RP, BG, LAB, EJC, PDC, and EGCL.

## Supplementary Material

Supplemental data

Unedited blot and gel images

Supporting data values

## Figures and Tables

**Table 1:**
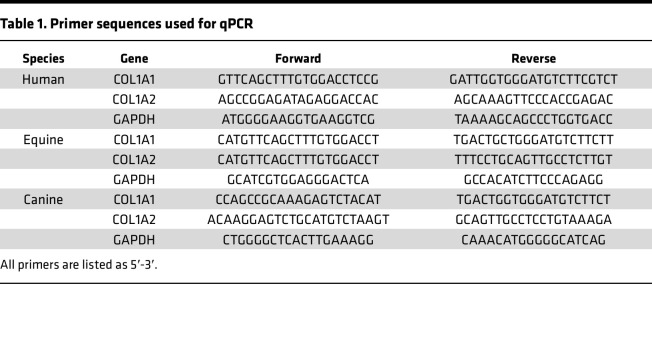
Primer sequences used for qPCR

## Version 1 05/08/2025

Electronic publication
