# Supplementary material for: Active synthesis of type I collagen homotrimer in Dupuytren’s fibrosis is unaffected by anti–TNF-α treatment
Source: JCI Insight. 2025 May 8;10(9):e175188. doi: 10.1172/jci.insight.175188 (PMC12128996; doi:10.1172/jci.insight.175188)
Supplement: Supplemental data [file jciinsight-10-175188-s189.pdf]

Supplementary Figures

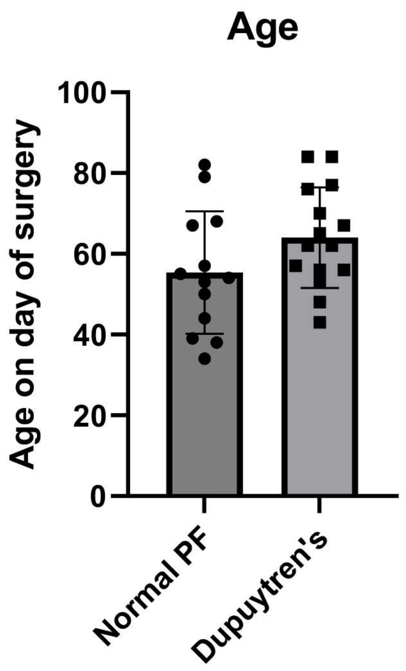

Figure S1: Age distribution of tissue samples used for qRT-pCR. No significant difference in the age of the patients on the day of surgery, when normal PF from carpal tunnel decompression (n=13) and Dupuytren's (n=15) samples were collected, was detected between the groups using a t-test.

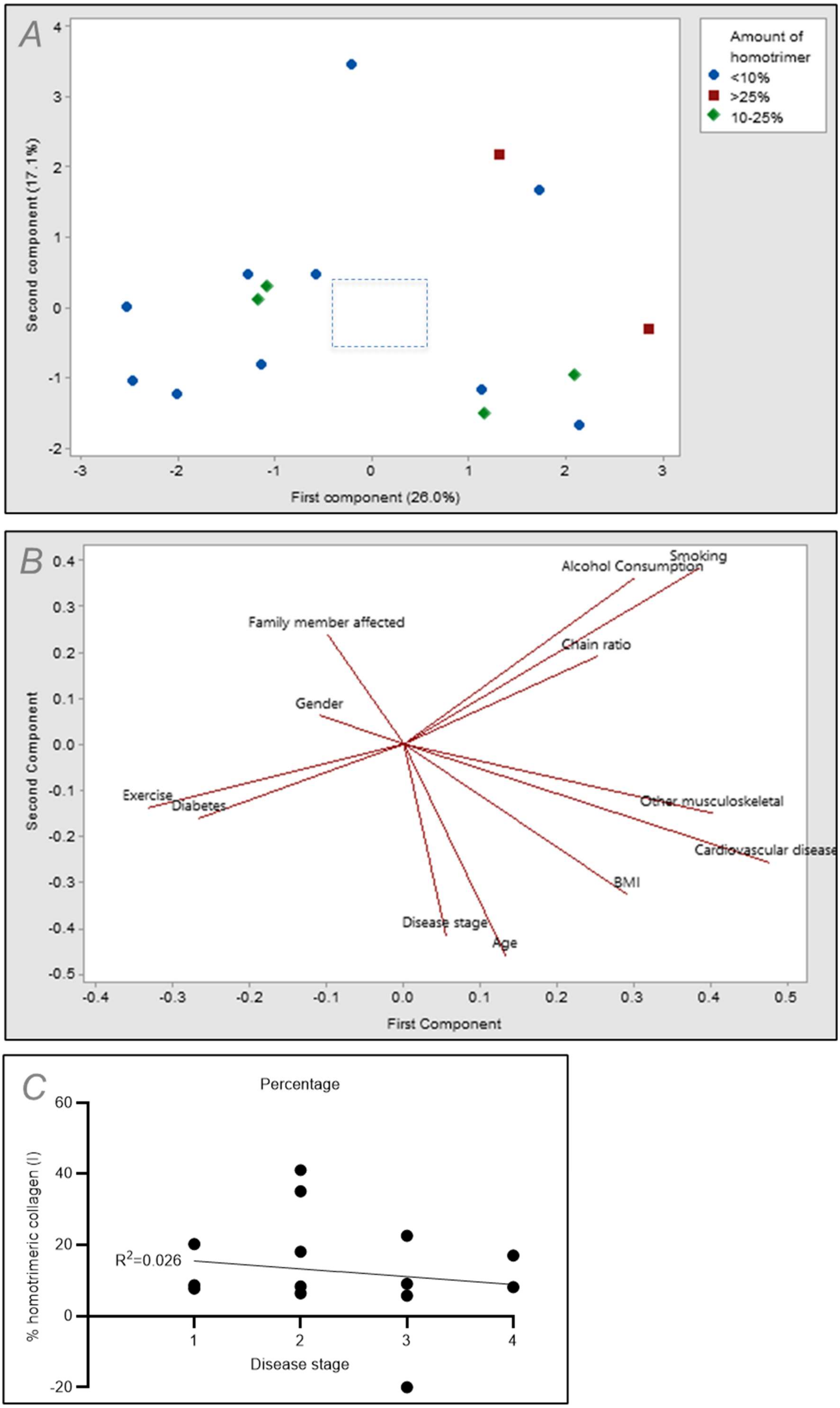
A
Amount of homotrimer
<10%
>25%
10-25%
Second component (17.1%)
First component (26.0%)
B
Alcohol Consumption
Smoking
Family member affected
Chain ratio
Gender
Exercise
Diabetes
Other musculoskeletal
Cardiovascular disease
BMI
Disease stage
Age
Second Component
First Component
C
Percentage
R^2=0.026
% homotrimeric collagen (I)
Disease stage

Figure S2: Principal components analysis of the relationship between demographic factors and the proportion of homotrimeric collagen synthesized by a sub-set of the human Dupuytren's surgical samples with disease stage information. A: Score plot grouped by lower (<10%), medium (10-25%) and higher (>25%) percentages of type I collagen homotrimer (n=16). The box indicates the relative scale on the loading plot (B). C: Plot of % homotrimeric collagen (I) versus disease stage reveals a very low inverse correlation (R^2 = 0.026).

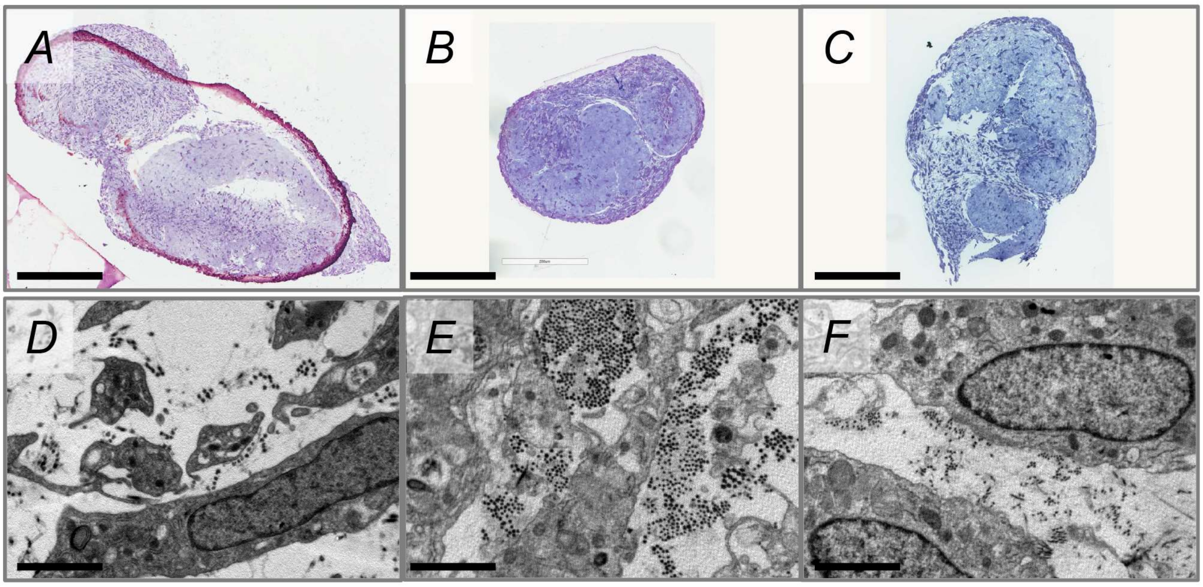

Figure S3: *De novo* collagen fibrils are present within tendon-like structures in 3D culture of normal palmar fascia and Dupuytren's cells. A-C: Semi-thin toluidine blue-stained sections of 3D tendon-like constructs (bar 200 um). D-F: Transmission electron microscopy images of 3D tendon-like constructs (bar 2 um). Constructs were derived from normal palmar fascia cells (A&D), Dupuytren's nodule (B&E) or Dupuytren's cord (C&F). Sample IDs are shown in Supplementary Table 3.

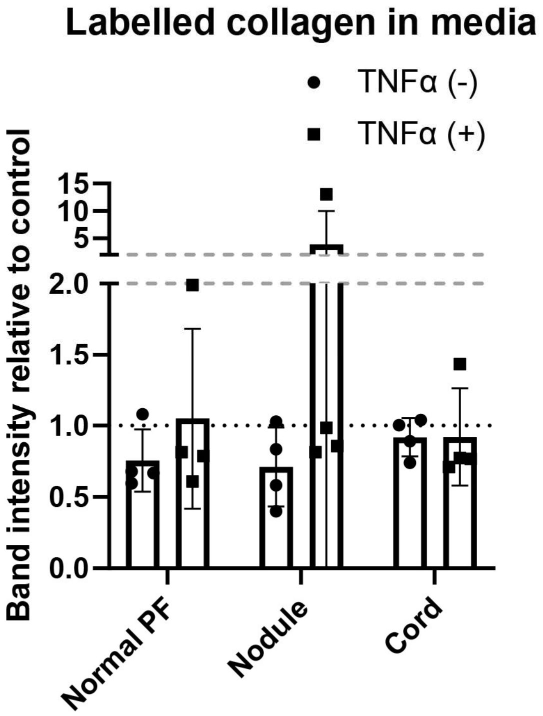

Figure S4. Comparison of the amount of labelled collagen in the media of tendon-like constructs treated with TNFα in the absence or presence of serum. Densitometric quantification of the relative amounts of radiolabelled (pro)collagen present in conditioned media from normal PF, Dupuytren's nodule and Dupuytren's cord cells (n=4) after TNFα treatment, as compared to control treatments, in serum free conditions (-) and in the presence of 10% FCS (+). Data are derived from Figures 6 I & L and were analysed by 2-way ANOVA.

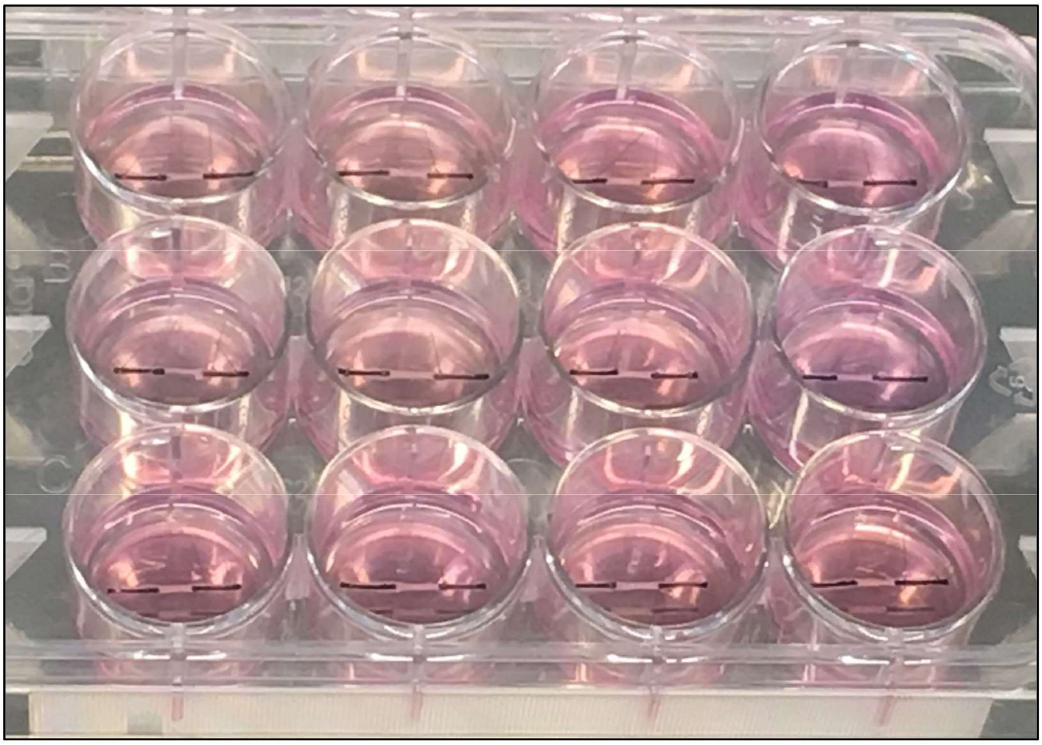

Figure S5. 3D tendon-like constructs formed in a 24 well plate. Image of a 24-well plate containing fully-formed 3D tendon-like constructs between pinned sutures (black) in each well.

Supplementary Tables

Supplementary Table 1: Dupuytren's human surgical samples used for collagen synthesis assays.

Sample ID	Age	Gender	Methods (pulse-chase: ^{14}C)	Demographics [A, B, C]	Data shown in Figure: (lane number)
140227-56-F	56	F	qPCR, ^{14}C	No	1A-G, D(12), 3A(12)
140227-81-M	81	M	^{14}C	-	1D(13), E-F, 3A(13)
140310-67-M	67	M	qPCR, ^{14}C	+	1A-G, D(14), 2A-D, 3A(14)
140310-76-M	76	M	qPCR, ^{14}C [D]	+	1A-G, D(15,16), 2A-D, 3A(15,16)
140312-43-M	43	M	qPCR, ^{14}C	+	1A-G, D(17), 2A-D, 3A(17)
140318-48-M	48	M	qPCR, ^{14}C	No	1A-G, D(18), 3A(18)
140320-56-M	56	M	qPCR, ^{14}C	+	1A-G, D(19), 2A-D, 3A(19)
140327-68-F	68	F	^{14}C	-	1E-F, 2A-B
140331-71-M	71	M	^{14}C	+	1E-F, 2A-D
140331-76-M	76	M	^{14}C [E]	-	1E-F, 2A-B
140423-68-M	68	M	^{14}C	+	1E-F, 2A-D
140423-69-M Nodule	69	M	qPCR, ^{14}C	+	1E-F, G, 2A-D
140512-47-M	14	M	^{14}C	+	1E-F, 2A-D
140528-52-M Nodule	52	M	qPCR, ^{14}C	+	1E-F, G, 2A-D
140529-58-M	58	M	^{14}C [F]	-	1E-F
140714-84-M	84	M	qPCR	No	1A-C
140804-71-M	71	M	^{14}C	+	1E-F, 2A-D
140804-84-M	84	M	qPCR	No	1A-C
140811-50-M Nodule	50	M	qPCR, ^{14}C	+	1E-F, G, 2A-D
140929-57-M	57	M	qPCR	No	1A-C
141006-67-M Cord	67	M	qPCR, ^{14}C	+	1E-F, G, 2A-D
141016-66-M	66	M	^{14}C	-	1E-F, 2A-B
141016-76-M	76	M	^{14}C	-	1E-F, 2A-B
141103-62-M	62	M	qPCR, (^{14}C [G])	No	1A-C, D(20), 3A(20)
141113-65-M	65	M	^{14}C [H]	-	1D(21,22), E-F, 2A-B, 3A(21,22)
141120-65-M	65	M	qPCR, ^{14}C	-	1A-G, D(23), 2A-B, 3A(23)
141120-80-F	80	F	^{14}C	-	1D(24), E-F, 2A-B, 3A(24)
150319-70-F	70	F	(^{14}C [I])	-	1D(25)
150611-62-F	62	F	qPCR	No	1A-C
150618-61-M-1 Nodule	61	M	^{14}C [J]	+	1E-F, 2A-D
150819-77-M	77	M	qPCR, (^{14}C [G])	No	1A-C
150910-53-M	53	M	qPCR, ^{14}C [K]	+	1E-F, G, 2A-D
150917-52-M	52	M	qPCR, ^{14}C	+	1A-C, E-F, G, 2A-D
150950-70-M	70	M	qPCR	No	1A-C
151015-72-M Nodule	72	M	qPCR, ^{14}C [L]	+	1E-F, G, 2A-D
160618-78-M	78	M	(^{14}C [G])	-	NA

Table footnotes: A: ‘No’ indicates no demographics information. B: ‘-‘ indicates demographics without disease stage. C: ‘+’ indicates that demographics include disease stage. D: Sample from thumb (16) was sufficiently labelled for quantification, sample from little finger (15) was not. E: Sample from thumb was sufficiently labelled, samples from ring and little finger were not. F: Mean value for cord and nodule utilised for quantification. G: Not sufficiently labelled for quantification. H: Mean value for cord (22) and nodule (21) utilised for analysis. I: Samples from neither the 4th (25) or 5th phalanges were sufficiently labelled for quantification. J: Cord was insufficiently labelled for quantification. K: Mean value for cord and nodule utilised. L: Cord was insufficiently labelled

Supplementary Table 2: Human normal Palmar Fascia (PF) surgical samples used for collagen synthesis assays.

Sample ID	Age	Gender	Methods (pulse-chase: ^{14}C)	Data shown in Figure: (lane number)
140723-82-F	82	F	qPCR, ^{14}C	1A-C, D(4), 3A(4)
140821-38-M	38	M	qPCR, ^{14}C	1A-C, D(2), 3A(2)
140821-39-F	39	F	qPCR, ^{14}C	1A-C, 3A(11)
140822-54-F	54	F	qPCR, ^{14}C	1A-C, D(3), 3A(3)
140822-55-F	55	F	qPCR, ^{14}C	1A-C, D(1), 3A(1)
140822-68-F	68	F	qPCR, ^{14}C	1A-C, D(6), 3A(6)
140911-48-M	48	M	^{14}C	1D(10), 3A(10)
141002-44-F	44	F	qPCR	1A-C, D(7), 3A(7)
141003-79-F	79	F	qPCR	1A-C
141007-57-F	57	F	qPCR	1A-C
141009-25-F	25	F	^{14}C	1D(8)
141013-34-M	34	M	qPCR	1A-C
141013-67-M	67	M	qPCR	1A-C
141103-51-F	51	F	^{14}C	1D(9)
141125-77-F	77	F	^{14}C	1D(5)
141128-50-F	50	F	qPCR	1A-C
141218-53-M	53	M	qPCR	1A-C

Supplementary Table 3. Human surgical samples used for cell culture and treatments.

Sample ID	Age	Gender	Tissue type	Data shown in Figure:
161012-62-F	62	F	Dupuytren's Cord & Nodule	4, 5 E& H(nodule), F,I(cord), 6 A-F(cord), G-H & J-K(nodule), I, L, 7 A&C(nodule), 7B
161109-70-M	70	M	Dupuytren's Cord & Nodule	4, 5B (nodule, 6-11), 6 A-F(cord), G-L, 7 A-C
161130-65-M	65	M	Dupuytren's Cord & Nodule	4, 5C (nodule, 12-17), 6 A-F(nodule), G-L, 7A-C
161130-76-M	76	M	Dupuytren's Cord & Nodule	4, 5A (cord, 1-4), 6 A-L, S3 B&E(nodule), D&F(cord), 7 A-C
170201-67-M	67	M	Normal Palmar Fascia	4, 6 A-F, I, L, 7B, D
170206-69-M	69	M	Normal Palmar Fascia	4, 6 I, L, 7B, D
170301-61-F	61	F	Normal Palmar Fascia	4, 5, 6 A-F, S3A, D, , 7B, D
170313-57-F	57	F	Normal Palmar Fascia	4, 5 D&G, 6 A-F, I, L, 7B, D
181126-71-M	71	M	Dupuytren's	8
190109-66-F	66	F	Dupuytren's	8
190313-63-M	63	M	Dupuytren's	8
190501-65-F	65	F	Dupuytren's	8, 9
190604-62-M	62	M	Dupuytren's	8, 9
190614-69-M	69	M	Dupuytren's	8, 9
170403-68-M	68	M	Dupuytren's Cord & Nodule	10
170403-86-M	86	M	Dupuytren's Cord & Nodule	10
170412-51-M	51	M	Dupuytren's Nodule	10
170412-68-F	68	F	Dupuytren's Nodule	10
170508-76-M	76	M	Dupuytren's Cord & Nodule	10
170515-65-M	65	M	Dupuytren's Cord & Nodule	10
170802-80-M	80	M	Dupuytren's Nodule	10
170816-73-F	73	F	Dupuytren's Cord	10
170816-78-M	78	M	Dupuytren's Nodule	10
