# Supplementary figures and images for: Active synthesis of type I collagen homotrimer in Dupuytren’s fibrosis is unaffected by anti–TNF-α treatment
Source: JCI Insight. 2025 May 8;10(9):e175188. doi: 10.1172/jci.insight.175188 (PMC12128996; doi:10.1172/jci.insight.175188)

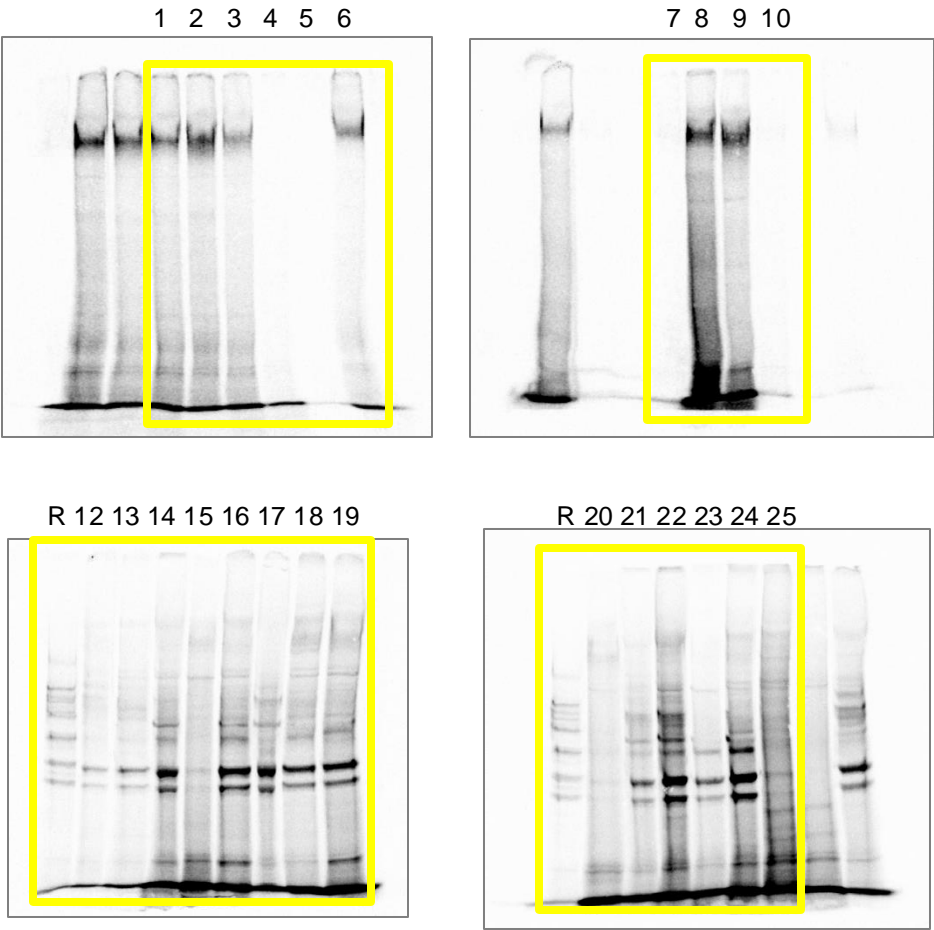

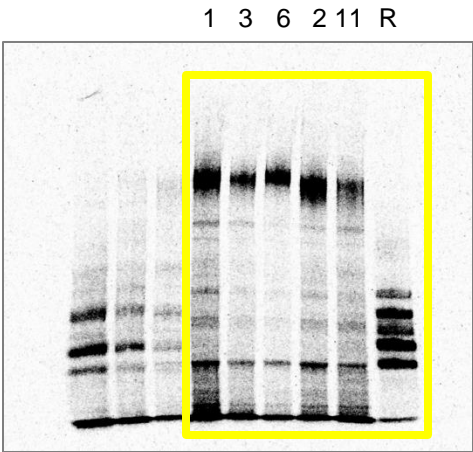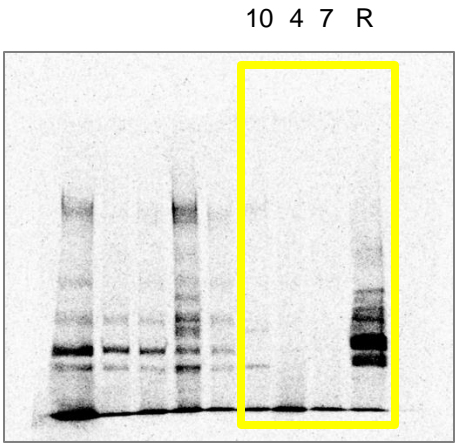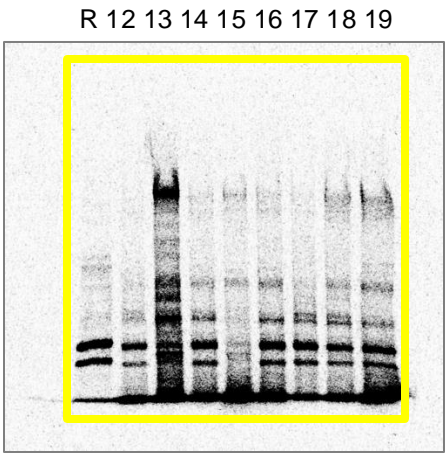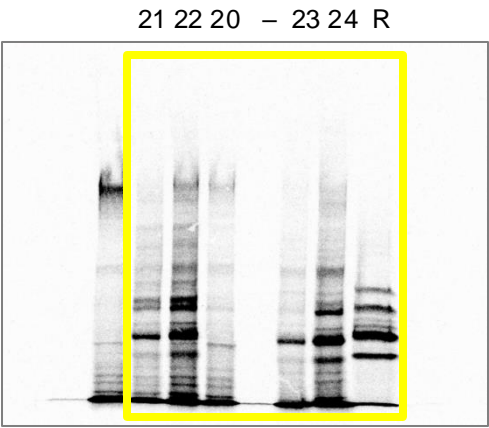

**E**    **Cranial cruciate ligament**  
         **Healthy**            **Ruptured**

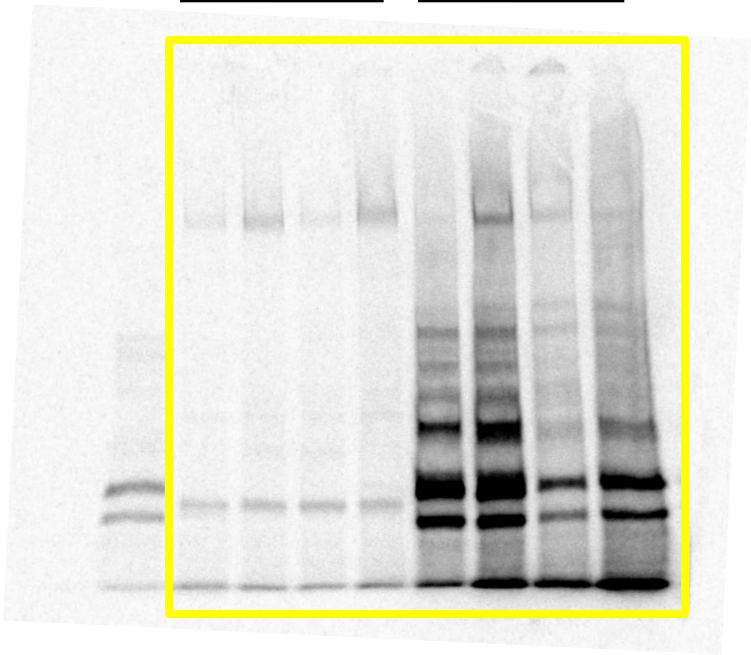

**F**    **Equine SDFT, age (yrs)**  
         **R <0 1 6 8 12 14 18 21**

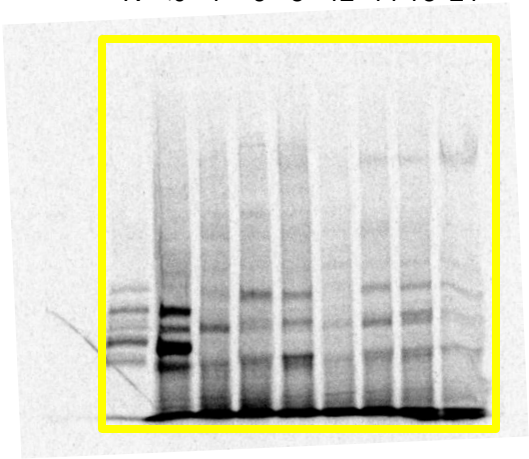

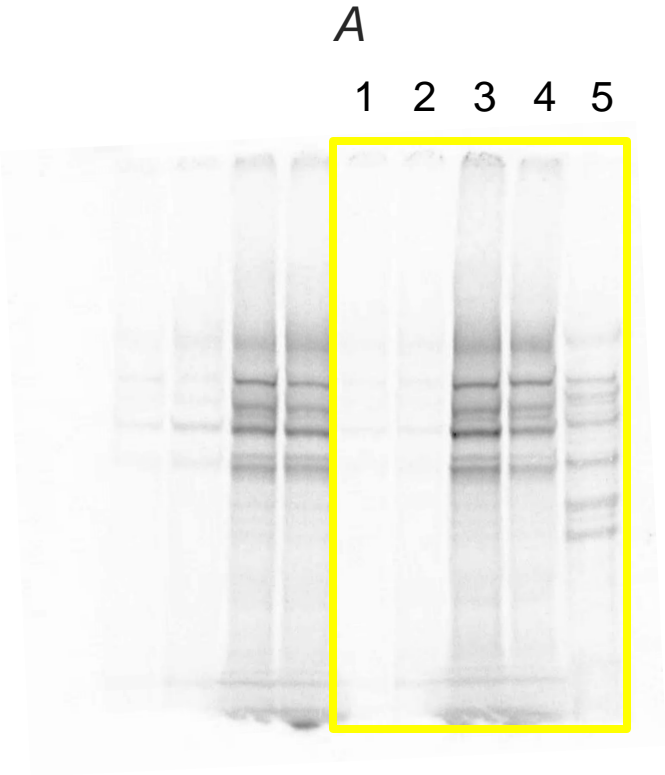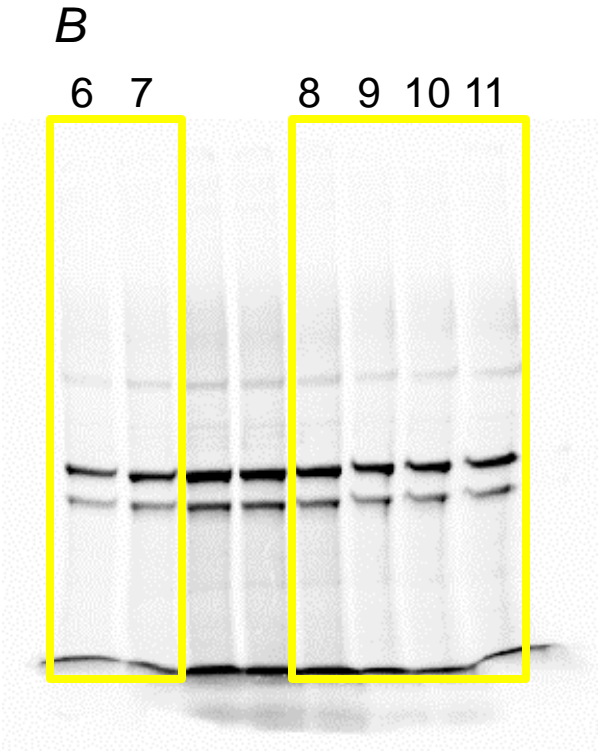

C

12 13

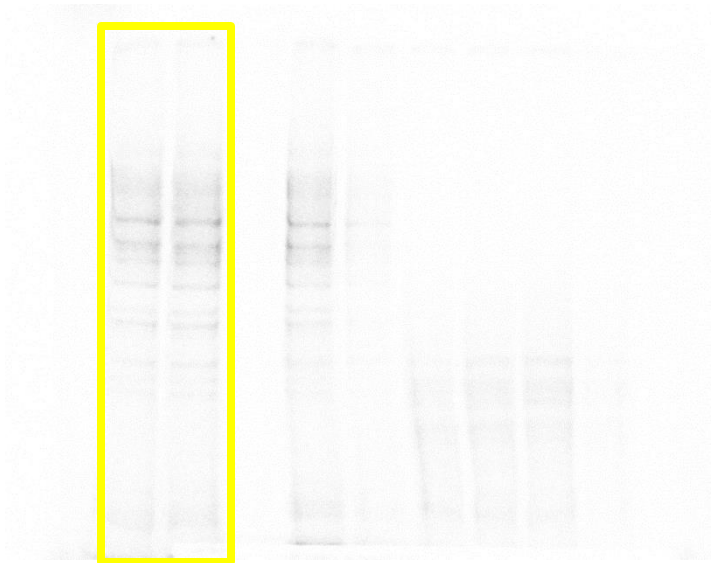

14 15

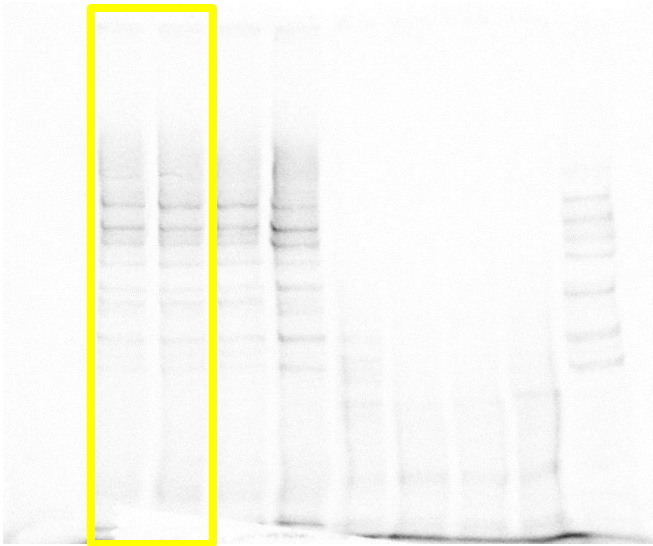

16 17

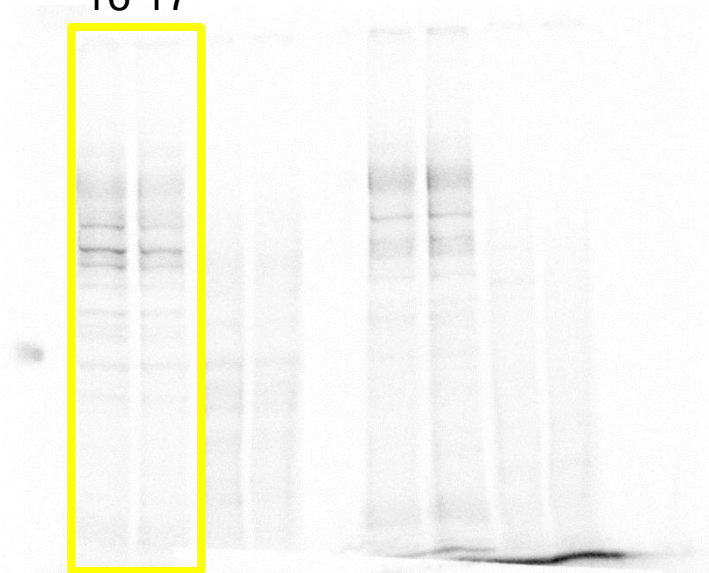

Supplement: Unedited blot and gel images [file jciinsight-10-175188-s190.pdf]
